# Optical control of Cas9 activity through visible-light cleavable crRNAs

**DOI:** 10.1039/d5cb00294j

**Published:** 2026-01-29

**Authors:** Vanessa Hanff, Stepan Jerabek, Kim A. Langer, Robin Klimek, Janik Kaufmann, Jimin Kim, Dieter Egli, Alexander Heckel

**Affiliations:** a Institute of Organic Chemistry and Chemical Biology, Goethe-University Frankfurt Max-von-Laue-Str. 9 Frankfurt am Main 60438 Germany heckel@uni-frankfurt.de; b Department of Pediatrics and Naomi Berrie Diabetes Center, Columbia University New York NY 10032 USA; c Columbia University Stem Cell Initiative New York NY 10032 USA; d Institute of Organic Chemistry and Biochemistry of the CAS Prague Czech Republic

## Abstract

In this work, we present several visible-light cleavable crRNAs targeting two different genes for regulating Cas9 activity. The crRNAs contain a total of three different photocleavable linker (PL) which induce a bond break when irradiated with a suitable wavelength. Cleavage of the crRNAs efficiently inhibits Cas9 activity, as demonstrated by the *in vitro* Cas9 assay developed in this work. By using visible light, our approach will enable precise downregulation of Cas9 activity in terms of location and timing, thereby enabling non-invasive and harm free control over genome editing for safer application.

## Abstract 2

Visible-light-cleavable crRNAs enable precise and non-invasive deactivation of Cas9 activity for safer genome editing.

## Graphical abstract

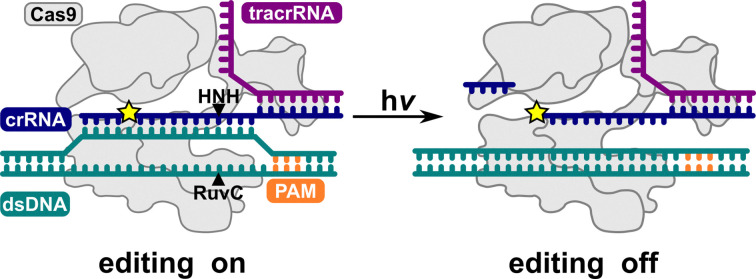

## Introduction

CRISPR/Cas9 is a powerful, Nobel Prize-winning tool in molecular biology that enables genome editing and gene regulation of living organisms. The principle was interpreted to be an antiviral “immune system” of bacteria.^1,2^ For the specific editing of a gene, either a single-stranded guideRNA or a duplex guideRNA consisting of a CRISPR RNA (crRNA) and a *trans*-activating CRISPR RNA (tracrRNA) is used to guide the Cas9 protein and derivatives thereof to its target locus. The latter must have both a proto-spacer adjacent motif (PAM) and be complementarity to the guideRNA. If this is the case, one strand of the target dsDNA can be displaced and a complex of DNA, Cas9, and the guideRNA can be formed. A conformational change in the Cas9 protein is followed by a double-strand break in the target dsDNA,^3,4^ which can then be repaired either by nonhomologous end joining (NHEJ) or by homology directed repair (HDR). The latter enables the targeted introduction of desired mutations using a template.^5^ The areas of application of Cas9 and modified proteins range from transcriptional regulation^6^ to gene editing,^7^ and it has already been used for therapeutic applications.^8,9^ In 2023, CASGEVY was also approved by the FDA as the first CRISPR/Cas9 gene therapy, now offering a cure for sickle cell anaemia, which until then had been considered incurable.^10^

Applications of Cas9 in research and therapy would benefit from precise spatiotemporal control. Even though CRISPR/Cas9 has been used more frequently in recent years as a cost-effective and accessible gene editing tool,^2,11^ negative effects such as unwanted off-target editing^12^ and genotoxicity^13^ can also be observed. In embryonic stem cells, it has also been shown that phenomena such as chromosomal aneuploidies can be associated with CRISPR/Cas9.^14^ Extended activity of Cas9 within a cell can lead to negative processes such as genetic mosaicism at on and off target locations.^15^ Precise temporal and spatial control of Cas9 activity is therefore essential to ensure safe use and minimize negative effects.^16–18^ Also, a degradable guideRNA enables temporal control of gene regulation.

Light is an ideal tool for this purpose as it offers remarkably high spatial and temporal resolution and is also a non-invasive method that is harmless when used correctly.^19,20^ Light-controlled regulation has therefore been studied in a wide variety of contexts,^21–23^ including Cas9 activity.^24,25^ Nihongaki *et al.* and Polstein *et al.* were able to design light-activated Cas9 proteins whose active form was released by irradiation with blue light, thus enabling targeted activation of editing.^26–28^ Rentmeister *et al.*, on the other hand, used “Flash-Caps” which they developed to photocage the mRNA of Cas9. This enabled them to achieve controlled activation of Cas9 translation *via* exposure to 365 nm light.^29^ In other approaches, the guideRNA was targeted for photoactivation rather than Cas9 itself. Deiters *et al.* showed that photocaging the Watson–Crick–Franklin side of the guideRNA led to complete inhibition of Cas9 activity, which could be effectively restored by irradiation with 365 nm.^17^ By using so-called blocking oligonucleotides, a similar effect could be achieved under irradiation.^30^ When unexposed, these can base pair with the guideRNA and thus prevent the formation of the CRISPR/Cas9 complex. They contain a 2-nitrobenzyl (NPE)-based photocleavable linker (PL), which allows cleavage by light and the base pairing with the guideRNA to be disrupted.^30^ In addition to the methods already described, there is also the option of a toehold displacement system. In this system, the guideRNA is extended at the 5' or 3' end by a toehold, which forces the guide sequence into a secondary structure and thus makes it unavailable to Cas9. An external DNA or RNA trigger displaces the toehold strand, making the guideRNA accessible again.^31,32^ Using various DNA triggers caged with NPE, Wang *et al.* succeeded in using this system to specifically regulate Cas9 activity.^33^

**Fig. 1:**
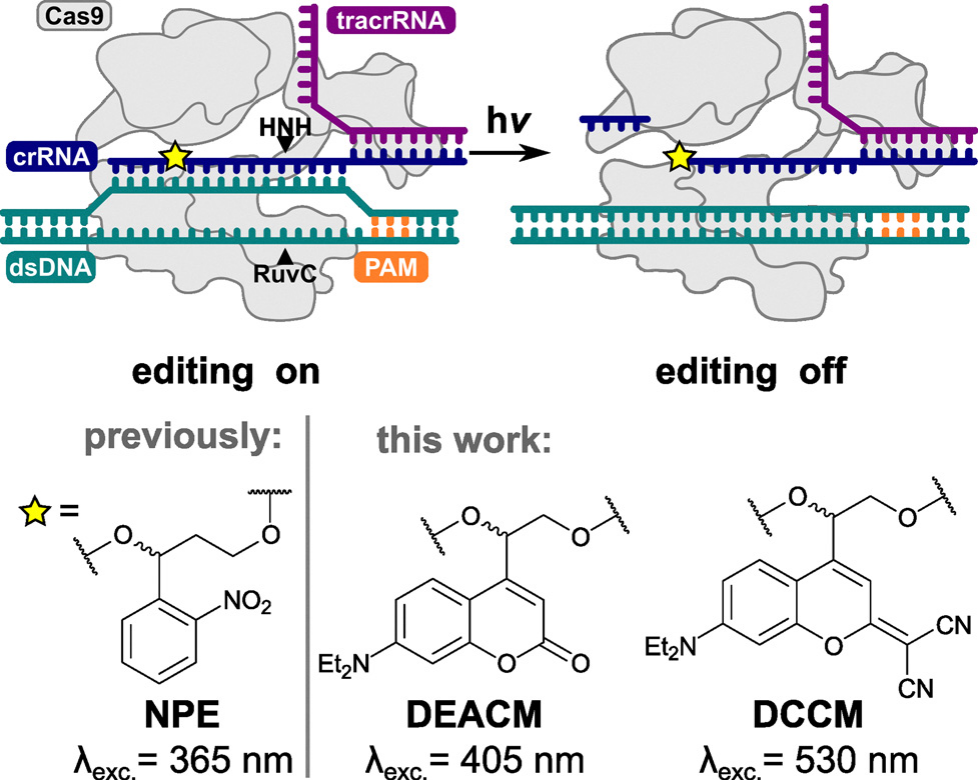
General principle of light-induced regulation of Cas9 activity by incorporating a photocleavable linker in the crRNA and the linkers that were used in this study.

While there are already several ways to turn Cas9 activity ON, the options turning Cas9 activity OFF with light are still very limited.^34–36^ Zou *et al.* addressed this gap by incorporating a NPE-based PL into the guide sequence of crRNAs for various target genes, such as MYC,^37^ whose overexpression is associated with cancer (Fig. 1, lower left).^34^ Irradiation of these photocleavable crRNAs led to a bond cleavage through the incorporated NPE. The remaining crRNA fragments no longer exhibit the required complementarity of 15 bp^38^ to the dsDNA and thus completely inhibit Cas9 activity.^34^ The demonstrated method currently needs UV light activation (365 nm). Prolonged exposure to light of this wavelength leads to oxidative stress in the cell and can potentially damage cell compartments. This is followed by a cascade of negative effects such as DNA damage, disruption of the cell cycle (see SI) and, in the worst case, even cell death especially in biological tissue.^39,40^

In this study, we present a set of novel photocleavable crRNAs, which in addition to the commercially available NPE PL, contain coumarin-based linkers with absorption maxima in the visible range of the electromagnetic spectrum (Fig. 1). As target genes for this study, we chose MYC and EYS, which play important roles in the progression of the cell cycle and the formation of the photoreceptor layer of the retina^37,41^ and are therefore two biologically extremely relevant genes. Furthermore, mutation and overexpression of these genes are associated with diseases such as retinitis pigmentosa and cancer.^42,43^ Precise yet safe editing, especially when used *in vivo*, is therefore highly desirable. By varying the position of the PL within the crRNA, we were able to find suitable positions that do not interfere with Cas9 activity in the unexposed state (ON state). However, irradiation with a suitable wavelength completely inhibited this activity (OFF state). Our method therefore not only provides a non-invasive and irreversible way of regulating Cas9 activity but also promises the possibility of editing without phototoxic UV light. This opens much safer handling of this powerful tool and contributes to the future of gene editing.

## Results and discussion

### Synthesis of the photocleavable linker

**Fig. 2:**
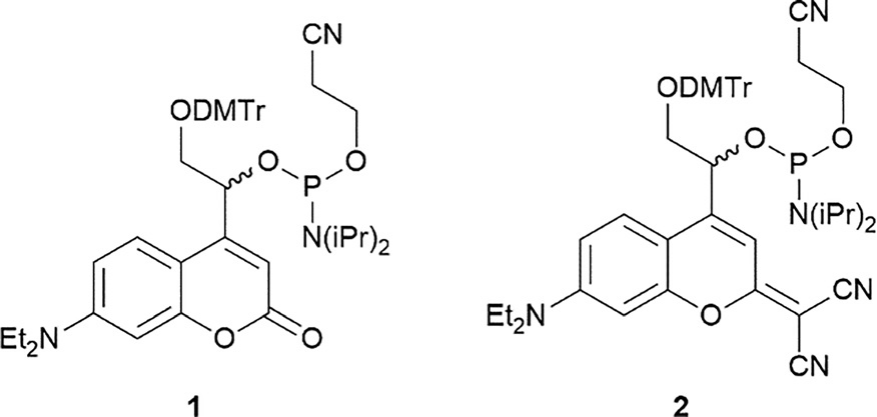
Chemical structures of the DEACM (**1**) and DCCM phosphoramidites (**2**).

To produce the visible-light cleavable crRNA, the required PLs first had to be synthesized. We selected a 7-diethylaminocoumarin (DEACM) derivative^44^ with *λ*_max_ = 391 nm and a dicyanocoumarin (DCCM) derivative^45^ with *λ*_max_ = 491 nm since their absorption maxima not only cover the blue but also the green spectral range. Furthermore, coumarins are characterized by their clean photochemical reaction, have already been incorporated into oligonucleotides,^44,45^ and their biocompatibility has already been demonstrated in *in vivo* applications.^46^ Also, we had previously demonstrated how this set can be used for chromatically selective photoactivation of one over the respective other.^47^ The phosphoramidites 1 and 2 (Fig. 2) were synthesized according to literature (see SI for full synthesis scheme).^44,45^

### Synthesis of the light-cleavable crRNA

**Fig. 3:**
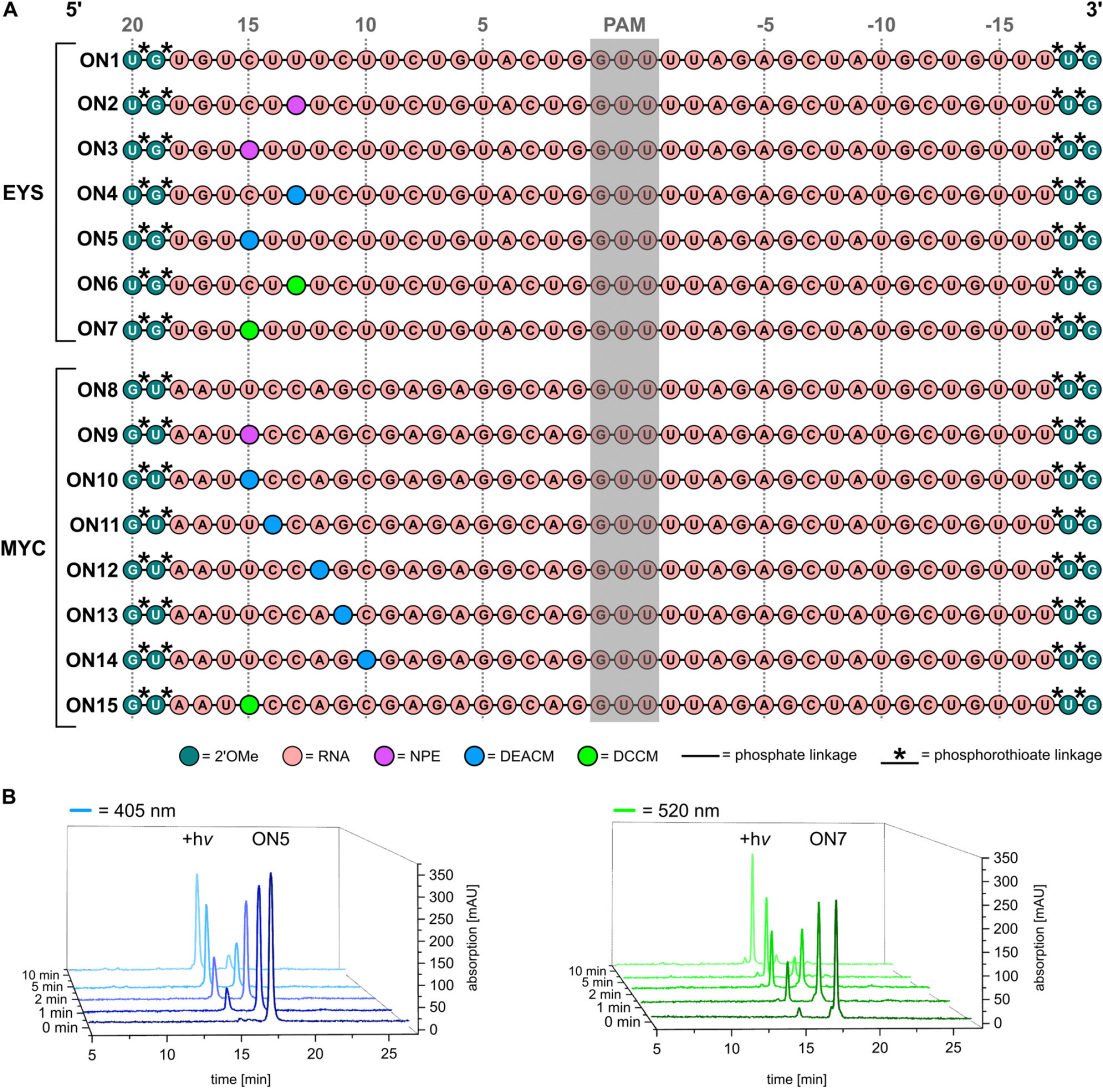
(A) Overview of the light cleavable crRNAs used in this study targeting for EYS and MYC with all modifications indicated. (B) **ON5** (left) and **ON7** (right) were chosen to demonstrate the exemplary uncaging of the synthesized crRNAs. 1.5 nmol of **ON5/ON7** were irradiated and 300 pmol were analysed *via* RP-HPLC. The resulting photo products were validated by mass spectrometry. Irradiation was performed with 405 nm and 530 nm Thorlabs LEDs (*I* = 700–1000 mA, *P* = 5–6 mW).

The synthesized PLs were then incorporated into the crRNA *via* solid-phase synthesis. The 42-nucleotide-long RNA constructs were modified at the 5′ and 3′ ends by two 2′-*O*-methyl RNA (2′-OMe) building blocks with phosphorothioate bonds to effectively protect the crRNAs against degradation by nucleases. A total of 15 different crRNAs were designed with 7 targeting EYS and 8 targeting MYC (Fig. 3A). For each target gene, a non-light-activated control was synthesized as well as various photocleavable constructs covering a total of three wavelengths (for detailed information on solid-phase synthesis, see SI). A special focus for the incorporation of the photocleavable linkers was placed on positions 15 and 13 (counting towards the 5′ end, starting at the PAM sequence). These were chosen because they are sufficiently distant from the PAM sequence and are therefore not involved in the formation of the so-called R-loop^1^ between dsDNA and crRNA.

This is essential for the function of CRISPR/Cas 9, and therefore modification and potential mismatches should be incorporated in regions further away from the PAM sequence whenever possible.^48^ The linkers were incorporated instead of a nucleobase, as no negative effect on the stability of the RNA/DNA duplex could be detected (see SI for melting point measurements). After successful synthesis of the various crRNAs, we investigated whether the desired bond cleavage occurs under irradiation. ON5 and ON7 were exemplarily investigated. 1.5 nmol of either oligonucleotide were irradiated with 405 or 530 nm light, respectively, and aliquots were analysed by RP-HPLC after the specified time intervals (Fig. 3B). A significant decrease of the intact crRNA (peak at ∼17 min) was observed. Instead, the corresponding photoproduct (peak at ∼15 min) appeared, which could be validated by mass spectrometry (see SI). Thus, 1.5 nmol of each oligonucleotide could be completely cleaved within 10 minutes of exposure to the corresponding wavelength.

### Activity of Cas9 with photocleavable crRNAs

After successfully demonstrating the desired light-induced cleavage of the crRNAs, an *in vitro* Cas9 assay was established. At first, the effectivity of double-strand breaks by Cas9 using the PL-containing crRNAs (ON state, before irradiation) with respect to the unmodified controls (ON1 and ON8) was compared to assess whether the introduction of a PL has a negative influence. After that, the extent to which the editing was abolished upon irradiation (OFF state) and the differences between performing these experiments in buffer and in cell lysate were investigated.

**Fig. 4:**
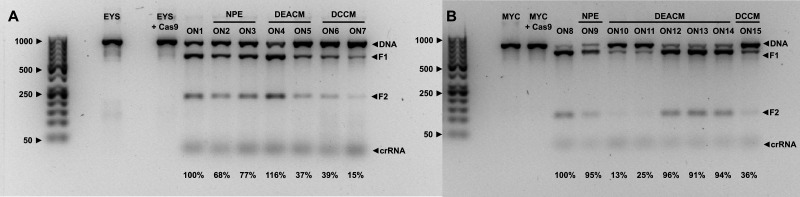
Cleavage efficiency for different crRNAs targeting for EYS (A) and MYC (B) in the ON state. 150 ng of DNA was used for each experiment. The protocol and details regarding the gel electrophoresis can be found in the SI.

To address the first question: the synthesized crRNAs were annealed with a universal tracrRNA at 95 °C for 5 minutes and cooled to room temperature. This guideRNA was then incubated with the Cas9 nuclease at room temperature for 30 minutes to ensure adequate complex formation between the two. The corresponding target dsDNA was then added. The ratio of dsDNA to guideRNA and Cas9 was 1 : 5 : 5 to enable the highest possible cutting efficiency. These were incubated for 1 hour at 37 °C (see SI for incubation times) and the reaction was stopped by adding proteinase K (55 °C for 15 minutes). The efficiency of Cas9 activity was then examined using agarose gel electrophoresis (Fig. 4, A for EYS and B for MYC). In addition, to the constructs to be tested, the respective dsDNA alone (EYS 869 bp and MYC 797 bp) and the dsDNA with Cas9 but without guideRNA were added to demonstrate the stability of the dsDNA under the conditions developed here.

With all (modified) guideRNAs, Cas9 was able to cut the dsDNAs, albeit with varying degrees of efficiency. This can already be seen in the non-light-activated controls ON1 and ON8. ON8 (targeting MYC) shows complete cleavage and both fragments (F1 and F2, see SI for determination of these) are clearly visible. ON1 (targeting EYS), on the other hand, still shows remaining uncut dsDNA. The sequence of the target DNA therefore appears to have an influence on the efficiency of cutting by Cas9. This is consistent with the data from Zou *et al.*, who also found different efficiencies for different genes. In their study, editing of the MYC gene was also the most effective.^34^ One explanation for this could be that MYC has a suitable PAM sequence 5′-AGG-3′ near the cleavage site. EYS, on the other hand, only has 5′-AGA-3′. This could contribute to the different efficiencies, as the PAM sequence is essential for Cas9 target recognition.^49^ The incorporation of modifications in the form of PLs also has an influence. The NPE constructs (ON2 and ON3 for EYS, ON9 for MYC) show similar behaviour to the corresponding non-light-activated controls. The PL was incorporated at positions 15 and 13, but its positioning within the sequence had no influence here. This changes when DEACM is used as the PL. The transition from a single-core aromatic system to a double-core system has a particularly negative effect on the cutting efficiency of Cas9 at position 15 (ON5 for EYS and ON10 for MYC), resulting in a significant amount of uncut dsDNA. However, if DEACM is incorporated at position 13 (ON4 for EYS), cleavage is in fact more efficient. This effect is even more pronounced the closer the PL is positioned to the PAM sequence. For ON12-14, the PL is located at positions 12, 11, and 10 counting from the PAM sequence. All three constructs show almost complete editing by Cas9, even outperforming the NPE construct ON9. This result thus deviates from the consensus in the literature, which recommends incorporating modifications in the PAM-distal region.^48^ The positioning of a modification therefore plays a major role in the efficiency with which Cas9 cuts and must be determined through screening. However, the type of modification also appears to play a significant role in the result. When DCCM is used as PL, the efficiency of Cas9 drops dramatically (ON6 and ON7 for EYS, ON15 for MYC). The cleavage products can only be detected weakly, and the amount of uncut dsDNA clearly predominates. This outcome is independent of the target gene and the position in the sequence.

Summarizing our findings to address our first question, Cas9-induced cleavage of two different target genes was demonstrated for crRNAs which contain coumarin-based PLs for the first time. Additionally, we showed that the efficiency with which this occurs depends largely on the sequence of the target DNA, the modification incorporated, and its positioning within the crRNA. To achieve the most complete editing possible, the interaction of these various influences must be determined and optimized through screening.

### Light-induced deactivation of Cas9 activity

**Fig. 5:**
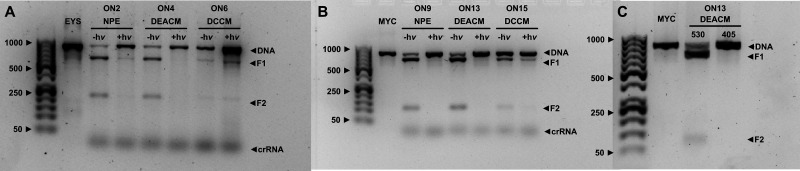
Light-induced downregulation of Cas9 activity (OFF state) for EYS (A) and MYC (B) using 150 ng dsDNA for each experiment. (C) Chromatically selective use of ON13 by first irradiating it with 530 nm and afterwards with 405 nm. Irradiations were performed with 365, 405 and 530 nm LEDs from Thorlabs (*I* = 700–1000 mA, *P* = 18–24 mW) for 10 min.

To address the second question – investigating if Cas9 activity can be turned OFF with light using our samples – one crRNA was selected for each PL (ON2, ON4, and ON6 for EYS and ON9, ON13, and ON15 for MYC). A Cas9 digestion was performed with a non-irradiated sample and compared with an irradiated sample (Fig. 5A and B). Irradiation was performed after incubation of the guideRNA with Cas9, before the dsDNA was added, using LEDs with wavelengths of 365 nm for NPE, 405 nm for DEACM, and 530 nm for DCCM.

The same result was observed for all three wavelengths and for both gels. In the unexposed state, the dsDNA was cleaved with efficiencies as described above. However, when exposed to light, complete inactivation of Cas9 activity was demonstrated for all PLs, and only uncut dsDNA could be detected. This is the first time that it has been shown that the principle of photocleavable crRNAs^34^ is transferable to PLs with excitation wavelengths in the visible range. In addition to the blue spectral range (λ_exc._= 405 nm), we also cover the green range (*λ*_exc._ = 530 nm). By using higher wavelengths, the damage caused by phototoxicity can be reduced.

In addition to the light-induced downregulation of Cas9 activity of the individual crRNAs, we were able to show that DEACM and DCCM can be used in a wavelength-selective manner^47^ (Fig. 5C). For this purpose, ON13 was used, which contains a DEACM at position 11 of the PAM sequence. This was first irradiated only with 530 nm, *i.e.*, with the wavelength used to excite DCCM. This did not lead to any light-induced bond breakage within the crRNA, and the activity of Cas9 was maintained. Only after additional irradiation with 405 nm could this be inhibited. This suggests that the targeted deactivation of two genes in a sequential order is possible if the crRNA for one gene contains DEACM and that for the other gene contains DCCM.

### Application of the *in vitro* Cas9 assay in HEK293T cell lysate

**Fig. 6:**
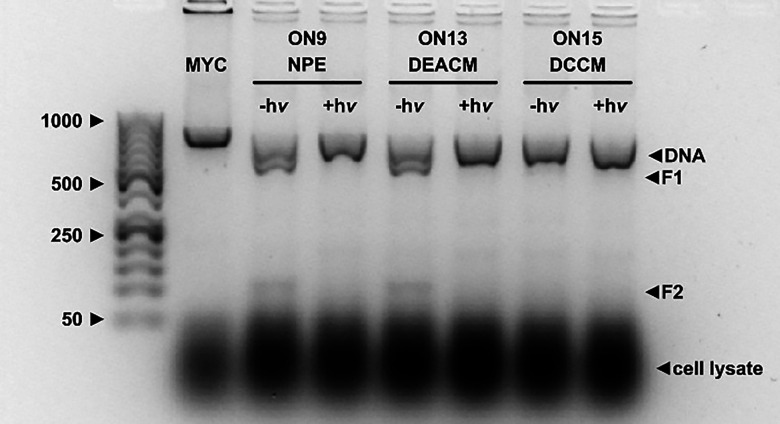
Application of the *in vitro* Cas9 assay to wt HEK293T cell lysate. Irradiations were performed with 365, 405 and 530 nm LEDs from Thorlabs (*I* = 700–1000 mA, *P* = 18–24 mW) for 10 min.

Next, we addressed our third question: to be able to use the *in vitro* Cas9 assay developed here *in vivo* in the future, it must be tested whether the parameters found are also applicable under similar conditions. Therefore, as an example, the dsDNA of MYC was dissolved in wild-type (wt) HEK293T cell lysate and a Cas9 digestion was performed with unirradiated and irradiated guide RNAs (ON9, ON13, and ON15, Fig. 6) as described above.

For crRNA containing NPE or DEACM, the same results as described above are obtained: almost complete cleavage in the unirradiated state, which can be completely switched off by irradiation with the appropriate wavelength. These constructs are therefore ideal candidates for future *in vivo* experiments. This contrasts with the DCCM construct. In previous experiments, this performed significantly worse than comparable DEACM constructs and showed only minor cleavage. However, when the assay is performed in cell lysate, Cas9 no longer cuts even in the unirradiated state. This difference between DEACM and DCCM, which both have the same coumarin scaffold is therefore unexpectedly large. This suggests that the dicyano groups have a greater influence on the activity of Cas9 than initially assumed. They may interfere with the formation of the complex between Cas9 and the target dsDNA or with the conformational change that Cas9 undergoes to perform the double-strand break. This could be verified and overcome by more intensive screening of the positioning analogous to DEACM to find a suitable candidate for *in vivo* experiments for DCCM as well.

## Conclusions

Over the past few years, CRISPR/Cas9 has developed into a versatile tool that has become indispensable in the field of genome editing. Since this technology was awarded the Nobel Prize, numerous therapeutic applications and even the first FDA-approved gene therapy based on this method have emerged. Despite these enormous strengths, the use of CRISPR/Cas9 carries risks in the form of off-target editing and genetic mosaicism, which is associated with excessive Cas9 activity in the cell. Precise temporal and spatial control over the duration of editing is therefore essential.

In this work, we were able to show that this can be achieved by positioning a visible light cleavable linker within the crRNA. By screening, we were able to identify positions in which Cas9 activity is not impaired in the unexposed state. However, irradiation with a suitable wavelength can completely inhibit this activity, as we were able to demonstrate in the *in vitro* Cas9 assay developed here. Unlike previous studies,^34–36^ we did not limit ourselves to commercially available NPE, but were able to show that this principle can also be applied to coumarin-based PLs for the first time. The associated shift of the activation wavelength into the visible range of the electromagnetic spectrum prevents phototoxic effects and thus contributes significantly to the safer application of this method in biological tissue. We have already succeeded in applying the construct developed here in HEK293T cell lysate and are therefore excited to see whether it can also be used in future *in vivo* applications.

## Author contributions

A. H., D. E. and S. J. conceived the project. A. H. supervised the study. V. H. designed and performed all experiments with support of K. A. L. during a bachelor thesis. R. K. and Ja. K. provided precursors for the synthesis of the phosphoramidites. Ji. K. performed the UVA exposure experiments. V. H. and A. H. wrote and revised the manuscript.

## Conflicts of interest

There are no conflicts to declare.

## Supplementary Material

CB-007-D5CB00294J-s001

## Data Availability

The data supporting this article have been included as part of the supplementary information (SI). Supplementary information is available. See DOI: https://doi.org/10.1039/d5cb00294j.
