# Supplementary material for: Optical control of Cas9 activity through visible-light cleavable crRNAs
Source: RSC Chem Biol. 2026 Jan 29;7(4):649–55. doi: 10.1039/d5cb00294j (PMC12908655; doi:10.1039/d5cb00294j)
Supplement: CB-007-D5CB00294J-s001 [file CB-007-D5CB00294J-s001.pdf]

Supplementary Information (SI) for RSC Chemical Biology.
This journal is © The Royal Society of Chemistry 2026

Supporting Information

Optical Control of Cas9 Activity through Visible-Light Cleavable crRNAs

Vanessa Hanff,[a] Stepan Jerabek,[b,c,d] Kim A. Langer,[a] Robin Klimek,[a] Janik Kaufmann,[a] Jimin Kim,[c] Dieter Egli[b,c] and Alexander Heckel*[a]

[a] *Institute of Organic Chemistry and Chemical Biology, Goethe-University Frankfurt, Max-von-Laue-Str. 9, Frankfurt am Main 60438, Germany.*
[b] *Department of Pediatrics and Naomi Berrie Diabetes Center, Columbia University, New York, NY 10032, USA.*
[c] *Columbia University Stem Cell Initiative, New York, NY 10032, USA.*
[d] *Institute of Organic Chemistry and Biochemistry of the CAS, Prague, Czech Republic*
Supplementary Information available: [details of any supplementary information available should be included here]. See DOI: 10.1039/x0xx00000x

Table of contents

1. Chemical synthesis of the phosphoramidites 2

1.1. General procedure 2

1.2. Full synthesis scheme 2

1.3. Synthesis of 2-(Bis(4-methoxyphenyl)(phenyl)methoxy)-1-(7-(diethylamino)-2-oxo-2*H*-chromen-4-yl)ethyl(2- cyanoethyl)diisopropylphosphoramidite (**1**) 3

1.4. Synthesis of 1-[2-(Dicyanomethylene)-7-(diethylamino)-2*H*-chromen-4-yl]-2-[(4-4’-dimethoxytrityl)oxy]ethyl(2-Cyanoethyl)diisopropylphosphoramidite (**2**) 4

2. Solid phase synthesis 5

2.1. General Procedure 5

2.2. Purification 6

2.3. Analysis 6

2.4. Irradiation of light-cleavable crRNAs 7

2.5. Duplex Melting Temperature Measurement 8

3. *In vitro* Cas9 Assay 9

3.1. General procedure and protocol 9

3.2. Sequences of dsDNA and cleavage fragments 10

3.3. Agarose gel electrophoresis 10

3.4. *HEK293T* Cell lysate 11

4. Effect of UVA light exposure on *HEK293T* cells 11

5. NMR Spectra 13

6. Mass spectra of the oligonucleotides and photo products 16

7. Analytical RP-HPLC of oligonucleotides 20

8. References 26

1. Chemical synthesis of the phosphoramidites

1.1. General procedure

Reactions were performed under exclusion of water using dry solvents and under a protective argon atmosphere. Dry solvents were purchased from *Acros Organics*, shipped and stored over molecular sieves. All reagents were purchased from commercial sources and used without further purification.

Silica gel 60-coated TLC sheets (ALUGRAM Xtra SIL G/UV, UV_{254} indicator) to monitor reactions as well as silica gel 60 (0.04-0.063 mm) for purification by column chromatography was purchased by *Macherey-Nagel*. In case of DMTR-protected compounds or phosphoramidites, the silica gel was deactivated by washing with the respective eluent containing 1% triethylamine (TEA).

NMR spectra were measured at room temperature (RT) on a *Bruker Avance III HD AV500 MHz* spectrometer. The residual solvent signals were used as reference (^{1}H: 7.26 ppm $CDCl_3$, 2.50 ppm DMSO-*d_6;* ^{13}C: 77.16 ppm $CDCl_3$, 39.52 ppm DMSO-d_6) and multiplicities are abbreviated as follows: s= singlet, d= doublet, t= triplet and m= multiplet. Coupling constants are reported in Hertz (Hz).

1.2. Full synthesis scheme

Starting with 7-(diethylamino)-4-(hydroxymethyl)-2H-chromen-2-one **S1**,[1] the alcohol group was converted to the corresponding chloride (→**S2**) with 4-methylsulfonic acid chloride. In a subsequent Wittig reaction with triphenylphosphine and formaldehyde, the alkene **S3** was formed, which could be oxidized to diol **S4** by Upjohn dihydroxylation with OsO_4 and N-methylmorpholine-N-oxide. For incorporation *via* solid-phase synthesis, the primary OH group was selectively tritylated with DMTr-Cl and then derivatized with 2-cyanoethoxy-N,N-diisopropylaminochlorophosphine to form the DEACM phosphoramidite **1**.[2] For the preparation of the DCCM phosphoramidite, starting from diol **S4**, the free OH groups were protected with TBDMS groups. Subsequently, the 2-position was substituted with sulfur and the dicyano group could be introduced using malonitrile and $AgNO_3$. After TBDMS deprotection, compound **S7** could be converted to DCCM phosphoramidite **2** in a manner analogous to the DEACM route (Scheme S1).[3]

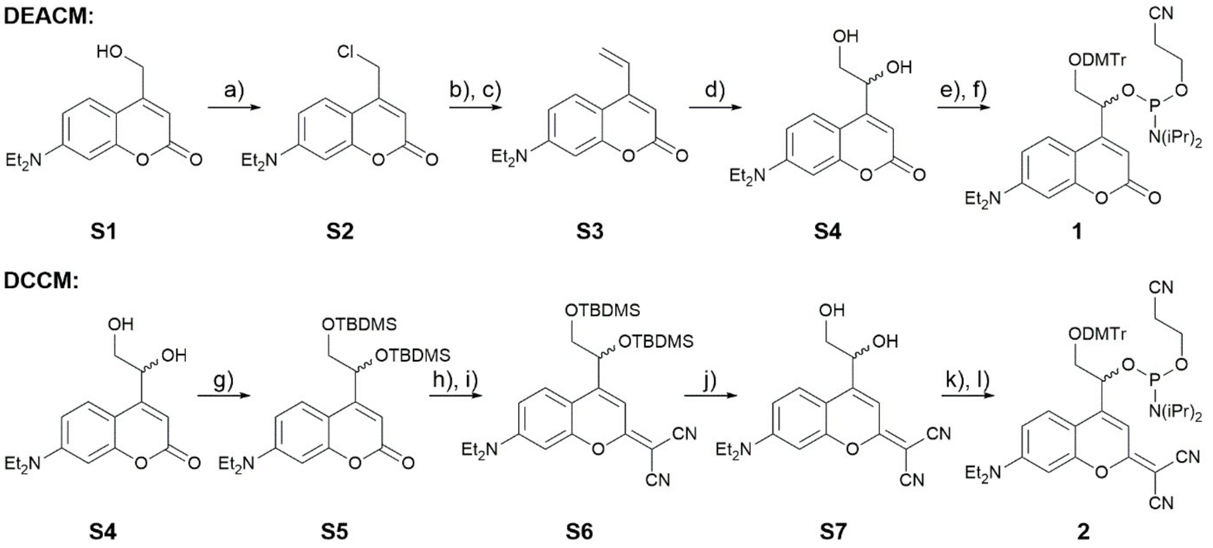

Scheme S1: Reagents and conditions: a) p-toluenesulfonyl chloride, 72 h, 42%; b) triphenylphosphine, 85 °C, 12 h, 75%; c) formaldehyde, 15 min, Na_2CO_3, quant.; d) OsO_4, 90 h, quant.; e) DMTr-chloride, 20 h, quant.; f) 2-cyanoethyl-N,N-diisopropylphosphorochloroamidite, DIPEA, 18 h, 90%; g) TBDMS-chloride, imidazole, 0 °C, 16 h, quant.; h) Lawesson's reagent, 110 °C, 9 h, 91%; i) malonitrile, $AgNO_3$, triethylamine, 3 h, 77%; j) acetic acid, TBAF, 0 °C, 16 h, 80%; k) DMTr-chloride, 0 °C, 16 h, 91%; l) 2-cyanoethyl-N,N-diisopropylphosphorochloroamidite, DIPEA, 18 h, 87%.

1.3. Synthesis of 2-(Bis(4-methoxyphenyl)(phenyl)methoxy)-1-(7-(diethylamino)-2-oxo-2*H*-chromen-4-yl)ethyl(2-cyanoethyl)diisopropylphosphoramidite (**1**)

All precursors were synthesized as previously described.[2]

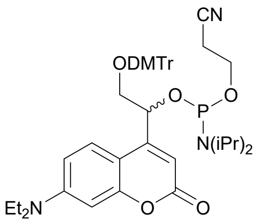

4-(2-(bis(4-methoxyphenyl)(phenyl)methoxy)-1-hydroxyethyl)-7-(diethylamino)-2H-chromen-2-one (1.00 g, 1.73 mmol, 1 eq) was dissolved in 30 mL dry DCM. DIPEA (1.76 mL, 10.35 mmol, 6 eq) was added and the mixture was stirred for 5 minutes. 2-Cyanoethoxy-*N,N*-diisopropylaminochlorophosphine (0.42 mL, 1.90 mmol, 1.1 eq) was added and the mixture was stirred for 16 h at room temperature. 20 mL saturated $NaHCO_3$ solution was added and the phases were separated. The aqueous phase was extracted twice with DCM. The combined organic phases were purified *via* column chromatography (cyclohexane/ethyl acetate 1:1). The product was dried under reduced pressure and obtained as a yellow solid.

Yield: 1.22 g (1.56 mmol, 90%)

R_f (cyclohexane/ ethyl acetate 1:1): 0.78

^{1}H NMR (500 MHz, DMSO-d_6):

δ= 7.47- 7.11 (m, 10H), 6.88- 6.74 (m, 4H), 6.59- 6.54 (m, 1H), 6.52- 6.51 (m, 1H), 6.07 (d, J= 2.6 Hz, 1H), 5.23- 5.20 (m, 1H), 3.84- 3.80 (m, 1 H), 3.72- 3.69 (m, 6H), 3.66- 3.55 (m, 2H), 3.46- 3.34 (m, 5H), 3.29- 3.17 (m, 1H), 2.76 (t, J= 5.9 Hz, 1H), 2.67 (t, J= 6.0 Hz, 1H), 1.17- 1.11 (m, 1H), 0.99 (d, J= 6.7 Hz, 1H), 0.86 (t, J= 6.9 Hz, 1H) ppm.

^{13}C NMR (126 MHz, DMSO-d_6):

δ= 161.21, 158.54, 156.45, 154.65, 150.69, 145.16, 135.82, 135.67, 130.13, 128.23, 128.07, 127.15, 119.37, 113.58, 108.98, 106.66, 105.84, 97.40, 86.25, 67.15, 59.16, 55.46, 44.41, 43.24, 31.42, 12.76 ppm.

^{31}P NMR (202 MHz, DMSO-d_6):

δ= 148.76, 148.66 ppm.

1.4. Synthesis of 1-[2-(Dicyanomethylene)-7-(diethylamino)-2*H*-chromen-4-yl]-2-[(4-4’-dimethoxytrityl)oxy]ethyl(2-Cyanoethyl)diisopropylphosphoramidite (**2**)

All precursors were synthesized as previously described.[3]

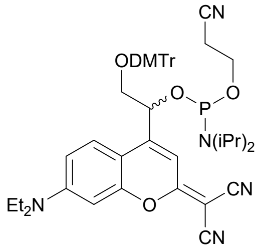

2-(4-(2-(bis(4-methoxyphenyl)(phenyl)methoxy)-1-hydroxyethyl)-7-(diethylamino)-2H-chromen-2-ylidene)malononitrile (400 mg, 637 µmol, 1 eq) was dissolved in 15 mL dry DCM. DIPEA (0.65 mL, 3.82 mmol, 6 eq) was added and the mixture was stirred for 5 minutes. 2-Cyanoethoxy-*N,N*-diisopropylaminochlorophosphine (0.16 mL, 701 µmol, 1.1 eq) was added and the mixture was stirred for 16 h at RT. 20 mL saturated $NaHCO_3$ solution was added and the phases were separated. The aqueous phase was extracted twice with DCM. The combined organic phases were purified *via* column chromatography (DCM/ MeOH 99:1). The product was dried under reduced pressure and obtained as an orange solid.

Yield: 500 mg (0.60 mmol, 94%)

R_f (DCM/MeOH 99:1): 0.43

^{1}H NMR (500 MHz, DMSO-d_6):

δ= 7.51- 7.42 (m, 1H), 7.32- 7.09 (m, 10H), 6.84- 6.70 (m, 6H), 6.63 (s, 1H), 5.41- 5.40 (m, 1H), 3.87- 3.75 (m, 1H), 3.70 (d, J= 1.8 Hz, 6H), 3.66- 3.64 (m, 3H), 2.76 (t, J= 5.9 Hz, 1H), 2.68- 2.61 (m, 1H), 1.22- 1.05 (m, 18H) ppm.

^{13}C NMR (126 MHz, DMSO-d_6):

δ= 171.69, 158.55, 155.23, 151.88, 145.12, 135.52, 130.08, 128.20, 127.94, 127.15, 119.45, 119.13, 113.48, 111.34, 107.43, 96.84, 86.06, 60.15, 55.45, 52.10, 44.67, 43.31, 24.84, 20.29, 12.73 ppm.

^{31}P NMR (202 MHz, DMSO-d_6):

δ= 149.02, 148.89 ppm.

2. Solid phase synthesis

2.1. General Procedure

RNAse-free water was used for oligonucleotides. All steps were performed under RNAse free conditions. Sterile pipette tips and sterile microreaction vessels were used. All surfaces and equipment were sterilized using 70% ethanol and RNase AWAY^{TM}-Spray from *Thermo Fisher Scientific*.

For solid phase synthesis, an *ABI 392 DNA/RNA synthesizer (Applied Biosystems)* at 1 µmol scale was used. 0.3 M 5-(benzylthio)-1H-tetrazole in acetonitrile (ACN) was used as activator. UltraMild capping reagents (Pac_2O in tetrahydrofuran/pyridine) and 3% TCA in DCM as detritylating reagent were purchased from *emp Biotech*. *Oxidizing (ABI)* by *J.T. Baker* was used for oxidation. Xanthane hydride (0.2 M in ACN/pyridine, *TCI)* was used as sulfurizing reagent.

UltraMild-protected phosphoramidites were used for all syntheses and purchased from *LGC Biosearch Technologies.* These include *rA(Pac), rC(Ac, rG(iPr-Pac), rU, 2'OMe-G(iPr-Pac)* and *2'OMe-U)*. They were used as 0.1 M solutions in dry ACN. The photolabile linkers **5** and **9** were used as 0.11 M solutions in dry ACN. A coupling time of 12 min was applied for all phosphoramidites. All oligonucleotides were synthesized in DMTr-Off mode.

As solid supports *2'OMe-G (dmf) SynBase CPG 1000/110 (LGC Biosearch Technologies), 2'OMe-G (nPac) CPG 1000 (ChemGenes*) and 3'-Spacer C3 (**S**) CPG (*LGC Biosearch Technologies*) were used.

Oligonucleotides containing NPE as a photolabile linker were purchased from *Biosynthesis*. The following oligonucleotides were synthesized as described above:

*Table S1: Overview of all synthesized sequences. mN = 2'OMe phosphoramidites, N= RNA phosphoramidites, *= phosphorothioate linkages.*

Name	Sequence (5' → 3')
ON1	mU*mG*UGUCUUUCUUCUGUACUGGUUUUAGAGCUAUGCUGUUU*mU*mG
ON4	mU*mG*UGUCU**[DEACM]**UCUUCUGUACUGGUUUUAGAGCUAUGCUGUUU*mU*mG
ON5	mU*mG*UGU**[DEACM]**UUUCUUCUGUACUGGUUUUAGAGCUAUGCUGUUU*mU*mG
ON6	mU*mG*UGUCU**[DCCM]**UCUUCUGUACUGGUUUUAGAGCUAUGCUGUUU*mU*mG***S**
ON7	mU*mG*UGU**[DCCM]**UUUCUUCUGUACUGGUUUUAGAGCUAUGCUGUUU*mU*mG* **S**
ON8	mG*mU*AAUUCCAGCGAGAGGCAGGUUUUAGAGCUAUGCUGUUU*mU*mG
ON10	mG*mU*AAU**[DEACM]**CCAGCGAGAGGCAGGUUUUAGAGCUAUGCUGUUU*mU*mG
ON11	mG*mU*AAUU**[DEACM]**CAGCGAGAGGCAGGUUUUAGAGCUAUGCUGUUU*mU*mG
ON12	mG*mU*AAUUCC**[DEACM]**GCGAGAGGCAGGUUUUAGAGCUAUGCUGUUU*mU*mG
ON13	mG*mU*AAUUCCA**[DEACM]**CGAGAGGCAGGUUUUAGAGCUAUGCUGUUU*mU*mG
ON14	mG*mU*AAUUCCAG**[DEACM]**GAGAGGCAGGUUUUAGAGCUAUGCUGUUU*mU*mG
ON15	mG*mU*AAU**[DCCM]**CCAGCGAGAGGCAGGUUUUAGAGCUAUGCUGUUU*mU*mG

After the synthesis, the cyanoethyl groups were removed with 20% diethylamine in ACN (*emp Biotech*) for 10 min to prevent formation of acrylonitrile adducts at the N^3 position of uridine bases. Cleavage from the solid phase was performed afterwards at 37 °C and 800 rpm with NH_4OH/EtOH (3:1, *Merck* and *Sigma Aldrich*) for 4 h in a thermal shaker (*Thermal Shaker Lite by VWR*). For oligonucleotides containing DCCM, 0.05 M K_2CO_3 in MeOH at RT for 4 h and 800 rpm was used. After spin filtration, the solvent was removed at 4 °C using a vacuum concentrator (*Speed Vac^{TM}, Thermo Fischer*). To remove the 2'-TBDMS-protecting groups, the oligonucleotides were redissolved in 115 µL DMSO (*Sigma Aldrich*). 60 µL TEA (*Sigma Aldrich*) and 75 µL TEA*3HF (*Sigma Aldrich)* were added. The mixture was incubated for 2.5 h at 55 °C and 500 rpm. After cooling to room temperature, 25 µL 3 M NaOAc in DEPC-H_2O and 1 mL -20 °C cold ethanol were added and the oligonucleotides were precipitated at -80 °C for 30 min or -20 °C over night. They were pelleted by centrifugation (*Centrifuge*

5415 R, Eppendorf) for 30 min at 4°C and 16.1 rcf. Residues of the solvent were removed at 4 °C using a vacuum concentrator.

2.2. Purification

Purification of the crude oligonucleotides *via* anion exchange chromatography was performed on an *ÄKTA Purifier System* with a *Resource™ Q polystyrene/divinylbenzene 16 mm x 30 mm column (Cytiva)*. Solvent A: 10 mM Tris, 25 mM $NaClO_4$, 20% ACN, pH 8, Solvent B: 10 mM Tris, 500 mM $NaClO_4$, 20% ACN, pH 8. Gradient 0-75% Buffer B in 1.5% per column volume at room temperature was used. The purified oligonucleotides were desalted using a 1 kDa cut-off membrane filter (*Microsep Advance Centrifugal Devices with Omega Membrane 1k, PALL*). Before adding the oligonucleotides, each filter was washed 5 times with DEPC-H_2O at 15000 g, 15 °C for 20 min. The desalting step was repeated 3 times.

After desalting, another purification step was performed on an *Agilent 1200 series* instrument equipped with a reversed phase *XBridge BEH C18 OBD Prep column (300 Å, 5 µm, 10 x 250 mm, waters)* at 60 °C. Solvent A: 400 nM hexafluoroisopropanol (HFIP, *fluorochem*) and 16.3 mM TEA (pH 7.8, *Sigma Aldrich*); Solvent B: MeOH (LiChrosolv™ from *Merck* or CHROMANORM™ from *VWR*). The following method was used with a flow rate of 3 mL/min:

Table S2: Method used for purification of the oligonucleotides.

Time [min]	% MeOH
0.00	5.00
1.00	5.00
8.50	20.0
23.50	32.0
25.50	100
27.50	100
30.00	5.00

Oligonucleotides containing DEACM were detected with an additional wavelength of 405 nm and DCCM with 520 nm. Residual solvent was removed at 4 °C using a vacuum concentrator. Afterwards, the oligonucleotides were desalted as described above.

2.3. Analysis

Analysis of all oligonucleotides was carried out at 40 °C *via* LC-MS (*Agilent 1200 system* equipped with *Waters Xbridge Peptide BEH C18 column (300 Å, 3.5 µm, 2.1mm x 250 mm)* in combination with a *Bruker micrOTOF-QII* ESI or a UPLC (*ThermoFisher Scientific Vanquish™ Flex* equipped with *Waters ACQUITY™ Premier Peptide BEH C18 (300 Å, 1.7 µm, 2.1 mm x 150 mm)* in combination with *ThermoFisher Scientific Orbitrap Exploris 120* mass spectrometer). The calculated and found mass of all oligonucleotides are listed below:

Table S3: Overview of the calculated and found mass for all synthesized oligonucleotides. The respective spectra can be found under chapter 6.

Name	Mass calculated [Da]	Mass found [Da]
ON1	13393.36	13392.03
ON4	13426.48	13426.56
ON5	13427.59	13427.06
ON6	13628.65	13628.05

ON7	13629.76	13629.91
ON8	13626.41	13624.91
ON10	13659.53	13658.01
ON11	13653.75	13659.97
ON12	13636.49	13636.80
ON13	13613.74	13617.49
ON14	13653.75	13658.61
ON15	13707.58	13707.75

Additionally, an analytical RP-HPLC run was performed with the same conditions as described for purification on a *Waters XBridge Peptide BEH C18 column (300 Å, 3.5 µm, 4.6 x 250 mm)* with a flowrate of 0.7 mL/min.

2.4. Irradiation of light-cleavable crRNAs

To show that irradiation of the light-activatable crRNA leads to the desired bond cleavage, **ON5** and **ON7** were selected as an example. The light-induced cleavage mechanism was elucidated for coumarin derivatives in 1999 by Schade *et al.*.[4] The chemical structures and sequences before and after light exposure of **ON5** are shown in Figure S1.

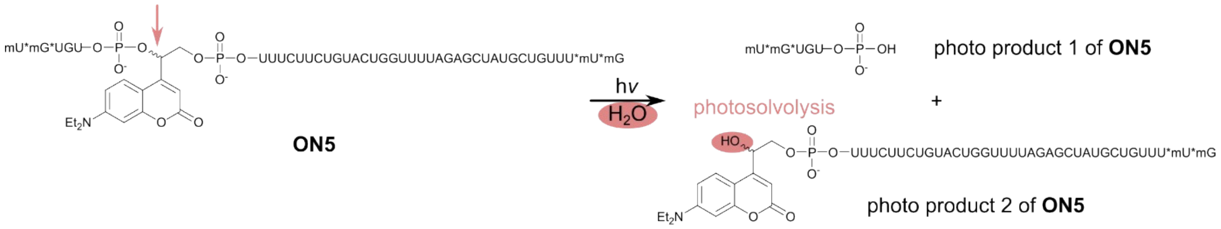

Figure S1: Photoreaction of ON5 with the chemical structure of the respective photo products formed upon irradiation. The cleavage side during photolysis is indicated with an orange arrow.

For the experiments 1.5 nmol of each oligonucleotide were dissolved in 400 µL DEPC-H_2O and placed under an LED of the corresponding wavelength. A 365 nm (*Thorlabs M365L2*), a 405 nm (*Thorlabs M405L2*) and 530 nm LED (*Thorlabs M530L3*) in combination with a *Thorlabs ACL2520-A* lens were used. After the indicated intervals (1, 2, 5 and 10 min), 300 pmol were analyzed *via* RP-HPLC as described in chapter 2.3. The new peak with an absorption at 405 or 520 nm was collected and identified and analyzed *via* LC-MS afterwards. The shorter photolysis product (5 nt) could not be detected.

Table S4: Overview of the possible photo products and their according mass. For the fragments showing an absorption at 405 or 520 nm, the according peak was collected and identified via mass spectrometry.

Name	Absorption at 405 or 530 nm?	Mass calculated [Da]	Mass found [Da]
Photo product 1 of **ON5**	-	1687.12	-
Photo product 2 of **ON5**	yes	11499.18	11498.47
Photo product 1 of **ON7**	-	1687.12	-
Photo product 2 of **ON7**	yes	11653.30	11651.64

2.5. Duplex Melting Temperature Measurement

Duplex melting temperatures were measured on a UV-vis spectrometer *Evolution 300* (*Thermo Fisher Scientific*) which was equipped with a Peltier element. 1 nmol of **ON1** and **ON4** were mixed with a fully complementary 42 nt DNA strand (purchased by *Biomers*) in 1 mL of a 1x PBS-buffered solution resulting in a 1 µM concentration. The spectra were recorded in a 10.00 mm path length quartz glass cuvette (*Hellma Analytics*). Each cycle consisted of a gradient with a slope of 1 °C/min from 20 °C to 85 °C and back to 20 °C. Eight cycles were performed, and the inflection point was determined as the melting temperature of each duplex. The resulting absorption spectra and melting points are shown in Figure S2.

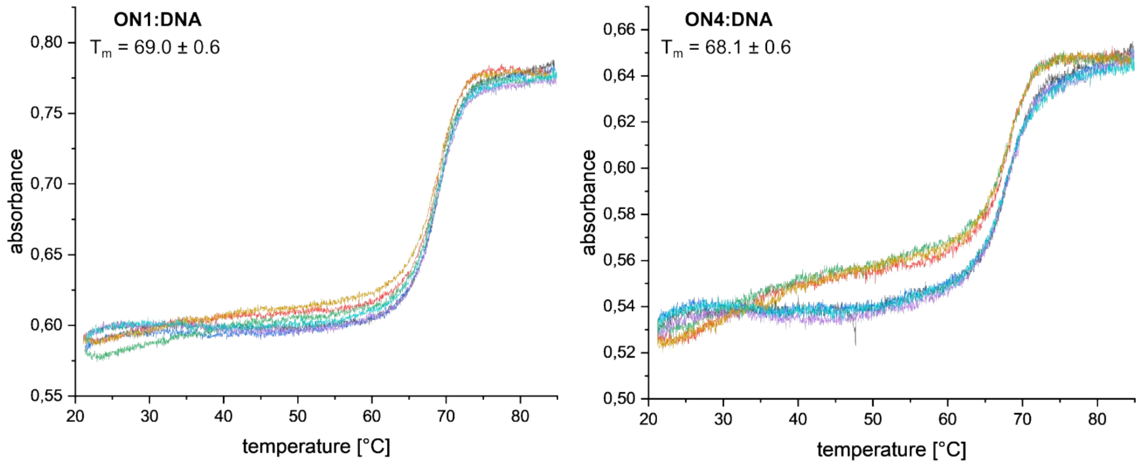

Figure S2: Melting temperature curves of ON1 and ON4 with a fully complementary 42 nt DNA strand (each ON 1 nmol in 1 mL 1x PBS buffer / 1 µM).

3. *In vitro* Cas9 Assay

3.1. General procedure and protocol

RNAse-free DEPC-H_2O was used for all reactions and buffers. All steps were performed under RNAse free conditions. Sterile pipette tips and sterile microreaction vessels or PCR tubes were used.

A *S. pyogenes* Cas9 nuclease was used for this assay, which was obtained from *New England Biolabs* as well as NEB Buffer 3.1 and Proteinase K. The universal tracrRNA was purchased from *IDT* and the included duplex buffer (30 mM HEPES, 100 mM potassium acetate, pH 7.5) was used. The dsDNA fragments were obtained as gBlocks™ by *IDT* as well.

For the results in the main paper, a total of 150 ng dsDNA was used according to the following protocol:

Preparation of the guideRNA:

- Assembly of the reaction in a PCR tube in the following order:

Component	Volume [µL]
Duplex buffer	28
100 µM tracrRNA	1.0
100 µM crRNA	1.0

- Annealed for 5 min at 90 °C and cooled to room temperature afterwards.
- Stored at -20 °C until use.

Cas9 enzyme digestion of dsDNA:

- Assembly of the reaction in a PCR tube in the following order:

Components	Volume [µL]
NEB Buffer 3.1	1.0
1 µM Cas9	1.5
Annealed guideRNA	0.5
Pre-incubate for 30 min at RT	
30 nM dsDNA	10

- Mixed thoroughly.
- Incubated at 37 °C for 1 hour.
- 1µL Proteinase K was added and mixed thoroughly.
- Incubated at 55 °C for 15 min.
- 2 µL loading dye was added to each sample and the fragments were analyzed *via* gel electrophoresis.

The probes were irradiated after the pre-incubation before the dsDNA was added.

3.2. Sequences of dsDNA and cleavage fragments

As targets for this assay, segments of the human EYS[5] (869 bp) and the human MYC gene[6] (797 bp) were used. The complement to the guideRNA is highlighted in blue, whereas the PAM sequence is highlighted in yellow. Cas9 cleaves 3 bp upstream beginning from the PAM sequence. The cleavage side is marked with "|".

Table S5: Sequences of the dsDNA used in this assay. The complementary of the guideRNA is highlighted in blue and the PAM sequence is highlighted in yellow. The length of the resulting fragments after cleavage of Cas9 were also determined and the indication is the same as in the main paper.

<table>
<tr><th>dsDNA</th><th>Length of the fragments [bp]</th><th>Sequence (5' → 3')</th></tr>
<tr><td>EYS</td><td>F1: 646
F2: 223</td><td>atgcttttatctctgagatacagtaccagccgatacattagcggcatgctttctgagttctgataactgtatcacagt
gtaatctctctgctcaggggtatgcagcctttcatttcttattccatttcaatttccctgtaggccagggaggggaaatt
ctacctagggagtttagcatcatttaacattgatgttactggtccaacaatctcctacatgagggagaccatatgtc
aaccacagttagcacttaaaatttgtcaatatctacttatacacatgaagctctttttattttgtgggaggtttaactttct
gaaagcattccatgtccaattttaaagtcagtaggagagaaggaaaataaaacgcagtgttagaaaagaaat
acatcttttatctacttgaaaatgtccacacttggctgggtccatatttcaggattgttgtgatccagaaagtacctctc
tcaatgaagaagtacttactatccatgtgtgaagttactcctttcttgccaaaaacacatatcataatggcctcttctg
ttttacgtttagctacacaacgcctgttggcagccctggagttgtttgtatgattgaaatgactgcagatggaaaac
ctccagtacagaagaaagaca | cagagatttcccatgcctctcaggtaggtatgcaaatatccagttccaaac
actaattgtgccctcagtatttcagataagtatttttatgttactaagcagtaatcatatttcttaattgaacagattcctt
gtaaaaactctcaacaaagcaccagaaaattcaactcatgctttagggatgattaaatatatttaatgttgcctgtc
actatcaaagtt</td></tr>
<tr><td>MYC</td><td>F1: 674
F2: 123</td><td>cctcgagaagggcagggcttctcagaggcttggcgggaaaaagaacggagggagggatcgcgctgagtat
aaaagccggttttcggggctttatctaactcgctgtagtaattccagcgagagg | cagagggagcgagcgggc
ggccggctagggtggaagagccgggcgagcagagctgcgctgcgggcgtcctgggaagggagatccgga
gcgaatagggggcttcgcctctggcccagccctcccgctgatcccccagccagcggtccgcaacccttgccgc
atccacgaaactttgcccatagcagcgggcgggcactttgcactggaacttacaacacccgagcaaggacgc
gactctcccgacgcggggaggctattctgcccatttggggacacttccccgccgctgccaggacccgcttctctg
aaaggctctccttgcagctgcttagacgctggatttttttcgggtagtggaaaaccaggtaagcaccgaagtcca
cttgccttttaatttattttttatcactttaatgctgagatgagtcgaatgcctaaatagggtgtcttttctcccattcctgc
gctattgacacttttctcagagtagttatggtaactggggctggggtgggggtaatccagaactggatcggggt
aaagtgacttgtcaagatgggagaggagaaggcagagggaaaacgggaatggtttttaagactaccctttcg
agatttctgccttatgaatatattcacgctgactcccggccggtcggacattcctgcttt</td></tr>
</table>

3.3. Agarose gel electrophoresis

For the analysis of the efficiency of the Cas9 cleavage, the reactions were analyzed *via* gel electrophoresis. Agarose was purchased from *Carl Roth* and was used as 2.5% solution unless stated otherwise. Tris-acetate-EDTA (TAE) buffer was used as a running buffer. A GeneRuler 50 bp DNA ladder (*Thermo Fisher Scientific*, includes loading dye) was used as reference.

For standard sized agarose gel, the *Standard Power Pack 25* by *Biometra* was used as a power supply in combination with a *SUB-CELL GT* gel electrophoresis tank from *Bio Rad*. For mini gels, a *PowerPac HV* power supply by *Bio Rad* in combination with a *Horizon 58* gel electrophoresis tank by *Whatman* were used. The voltage and duration were 50 V for 3 h for standard sized agarose gel and 50 V for 1.5 h for mini gel unless stated otherwise.

SYBR™ Gold DNA gel stain (10000x concentrate*, Thermo Fisher Scientific*) was used for staining, and the gels were imaged using a *Gel Doc™ XR+ molecular imager* by *Bio Rad*.

3.4. *HEK293T* Cell lysate

The wildtype (wt) *HEK293T* cell lysate was received from Fabian Sinsel and Marius Külp and was prepared as previously described.[7] The lysate was used to dissolve dsDNA resulting in a 30 nM solution.

Table S6: Number of cells and the viability before lysis for the cell lysate used in this assay.

Name	Cell count	Viability [%]
293T::wt 1	$3.17*10^6$	92%
293T::wt 5	$3.24*10^6$	93%

4. Effect of UVA light exposure on *HEK293T* cells

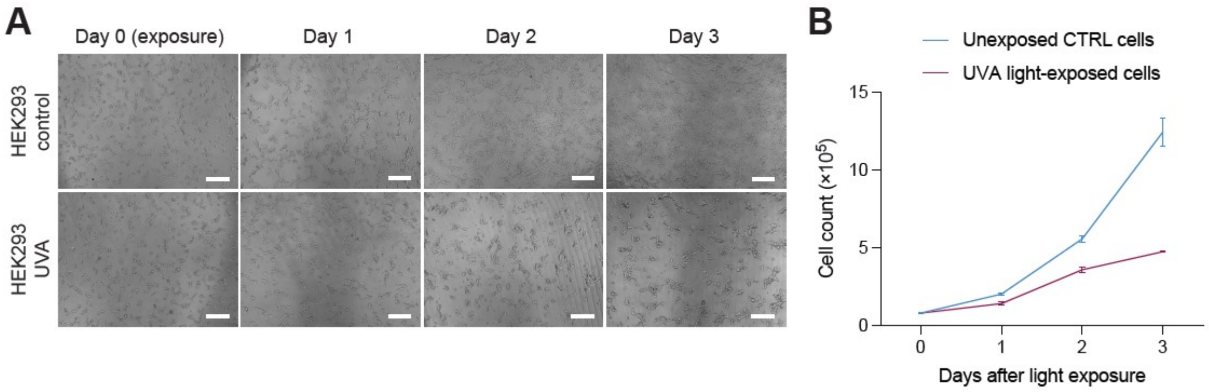

Figure S3: Treatment with UVA light has a negative effect on the proliferation of cultured human embryonic kidney (HEK293T) cells. A: Bright field images showing proliferation of human embryonic kidney (HEK293T) cells treated with UVA (365 nm) light for 1 minute compared to untreated control cells. Both cohorts were imaged at the time of the exposure and on each consecutive day during the three-day experiment. Scale bars= 200 µm. B: Growth curves for UVA-exposed HEK293 cells and unexposed control cells. Cells were counted on an automated cell counter with n = 3 replicates per time point and cohort.

Human embryonic kidney (HEK293T) cells were cultured in Dulbecco's Eagle Modified Medium (DMEM) with GlutaMAX (*Thermo Fisher Scientific*; Cat. number 10566016) supplemented with 10% fetal bovine serum (FBS) (*Sigma Aldrich*). The cells were cultured at 37 °C in a humidified incubator with 5% CO_2 and passaged every 4 days at confluency around 70%. One day prior to the experiment, cells were counted using the *Countess 3 Automated Cell Counter (Thermo Fisher Scientific*) and seeded at a density of 50 x 104 per well. The next day, half of the wells were exposed to the UVA light (365 nm) using an LED flashlight (*Jaxman*; Model U1c 3W) fixed at ~2 cm above the bottom of the well for 1 minute. Control wells with HEK293T cells were left unexposed. Both UVA-exposed cells and unexposed control cells were imaged using an inverted *Olympus IX73 microscope* and counted on each day during the experiment, lasting three days after the exposure. Collected data (Supplementary Table 7) were plotted in GraphPad Prism (Version 10.3.1).

Table S7: Collected Data of the growth of UVA light exposed HEK293T cells plotted in figure S1.

Day	Sample	Total Cell Number	Viability
DAY "-1": plating	All the same	50000	Only live cells counted
DAY 0: exposure	All the same	78000	95
	All the same	75000	94
	All the same	75000	93
	All the same	78000	92
DAY 1	CTRL (unexposed)	202500	93
	CTRL (unexposed)	210000	95
	CTRL (unexposed)	187500	91
	CTRL (unexposed)	195000	93
	UVA (365 nm; exposed 1 min)	127500	90
	UVA (365 nm; exposed 1 min)	150000	93
	UVA (365 nm; exposed 1 min)	135000	90
	UVA (365 nm; exposed 1 min)	142500	91
DAY 2	CTRL (unexposed)	540000	94
	CTRL (unexposed)	560000	96
	CTRL (unexposed)	540000	95
	CTRL (unexposed)	580000	96
	UVA (365 nm; exposed 1 min)	360000	89
	UVA (365 nm; exposed 1 min)	340000	88
	UVA (365 nm; exposed 1 min)	380000	85
	UVA (365 nm; exposed 1 min)	340000	90
DAY 3	CTRL (unexposed)	1152000	92
	CTRL (unexposed)	1224000	91
	CTRL (unexposed)	1368000	93
	CTRL (unexposed)	1224000	95
	UVA (365 nm; exposed 1 min)	472500	92
	UVA (365 nm; exposed 1 min)	466200	90
	UVA (365 nm; exposed 1 min)	472500	91
	UVA (365 nm; exposed 1 min)	478800	92

5. NMR Spectra

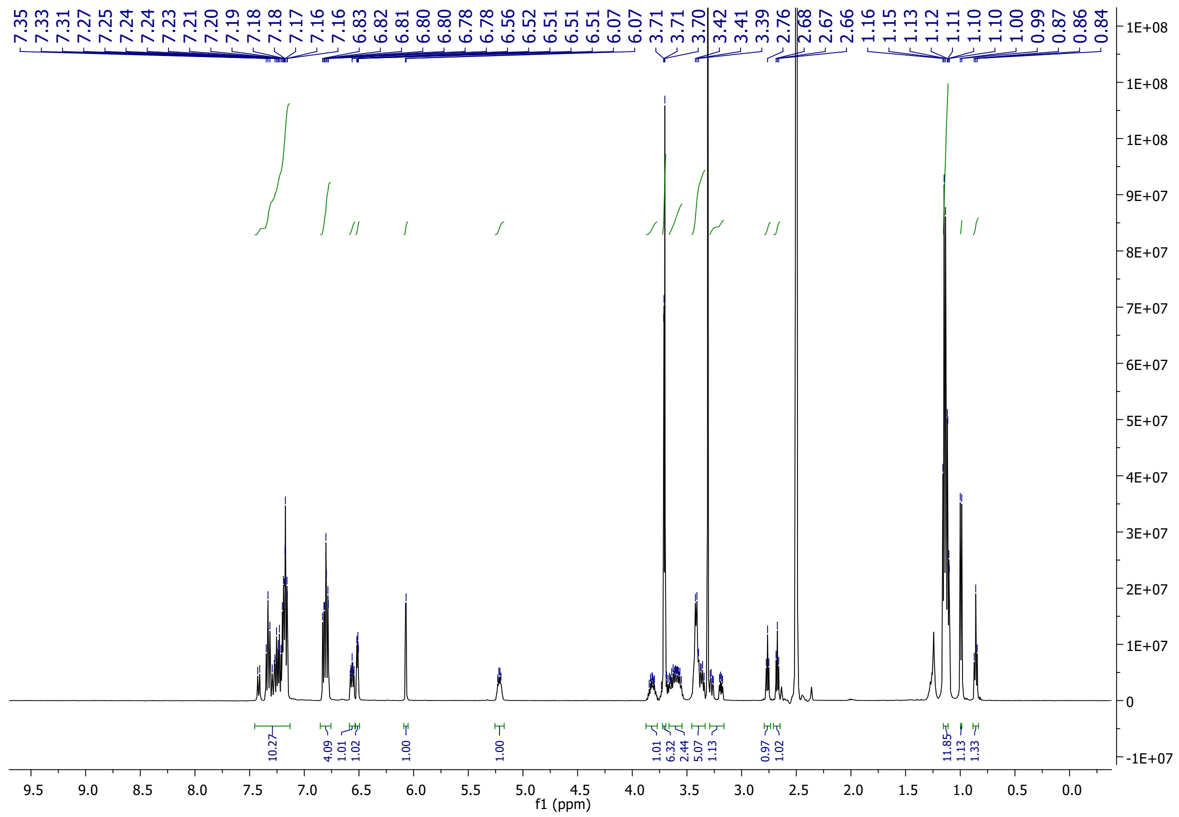

Figure S4: ^{1}H-NMR of 2-(Bis(4-methoxyphenyl)(phenyl)methoxy)-1-(7-(diethylamino)-2-oxo-2H-chromen-4-yl)ethyl(2-cyanoethyl)diisopropylphosphoramidite in DMSO. The NMR shows residues of water.

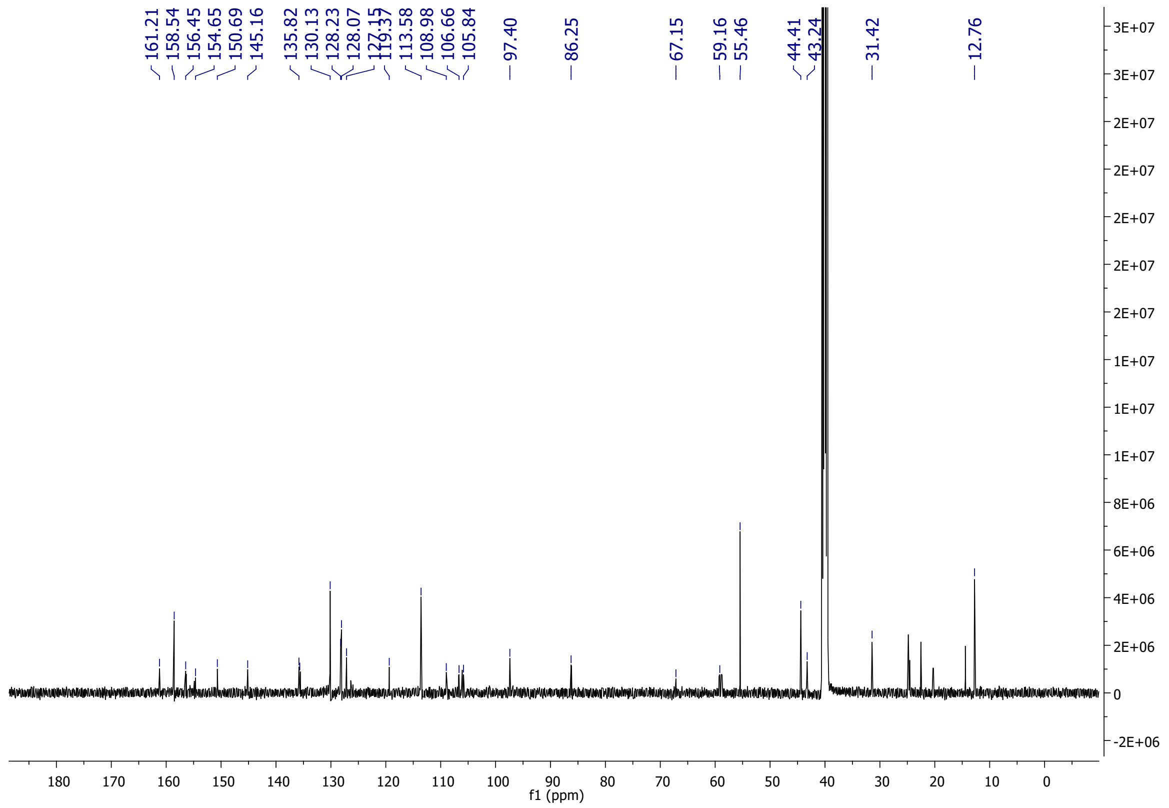

Figure S5: ^{13}C-NMR of 2-(Bis(4-methoxyphenyl)(phenyl)methoxy)-1-(7-(diethylamino)-2-oxo-2H-chromen-4-yl)ethyl(2-cyanoethyl)diisopropylphosphoramidite in DMSO.

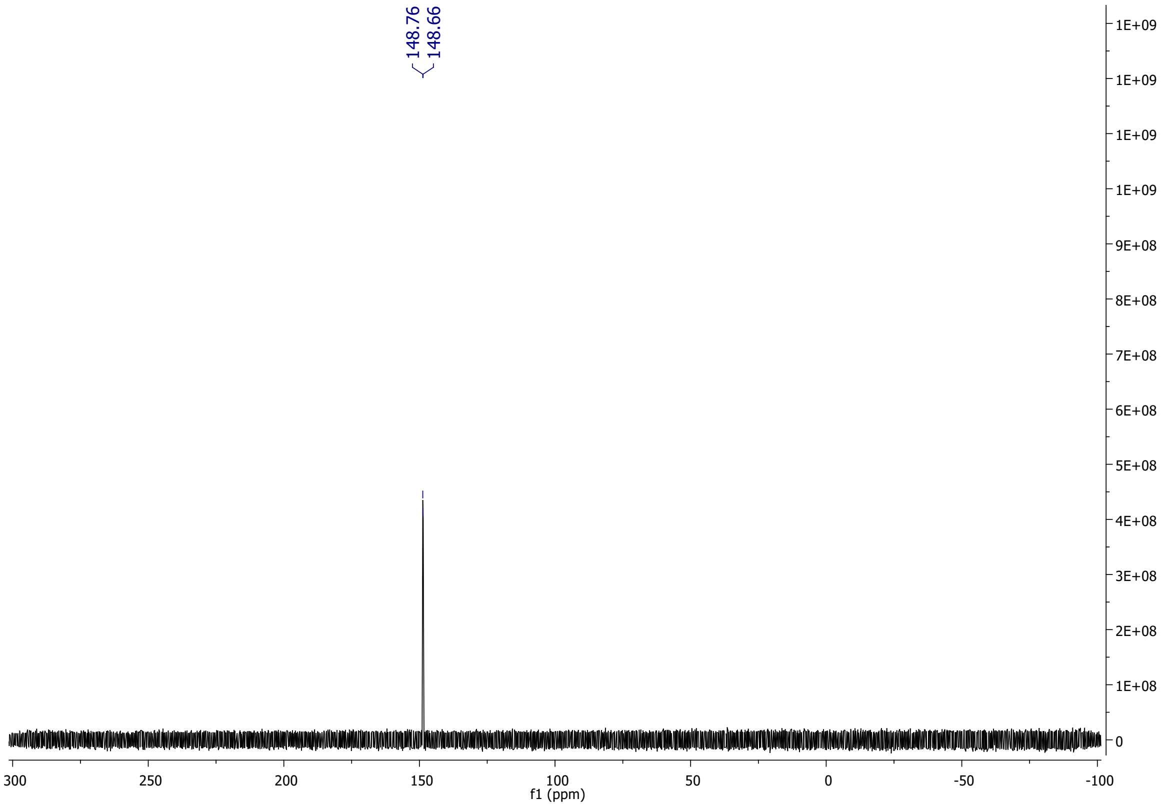

Figure S6: ^{31}P-NMR of 2-(Bis(4-methoxyphenyl)(phenyl)methoxy)-1-(7-(diethylamino)-2-oxo-2H-chromen-4-yl)ethyl(2-cyanoethyl)diisopropylphosphoramidite in DMSO.

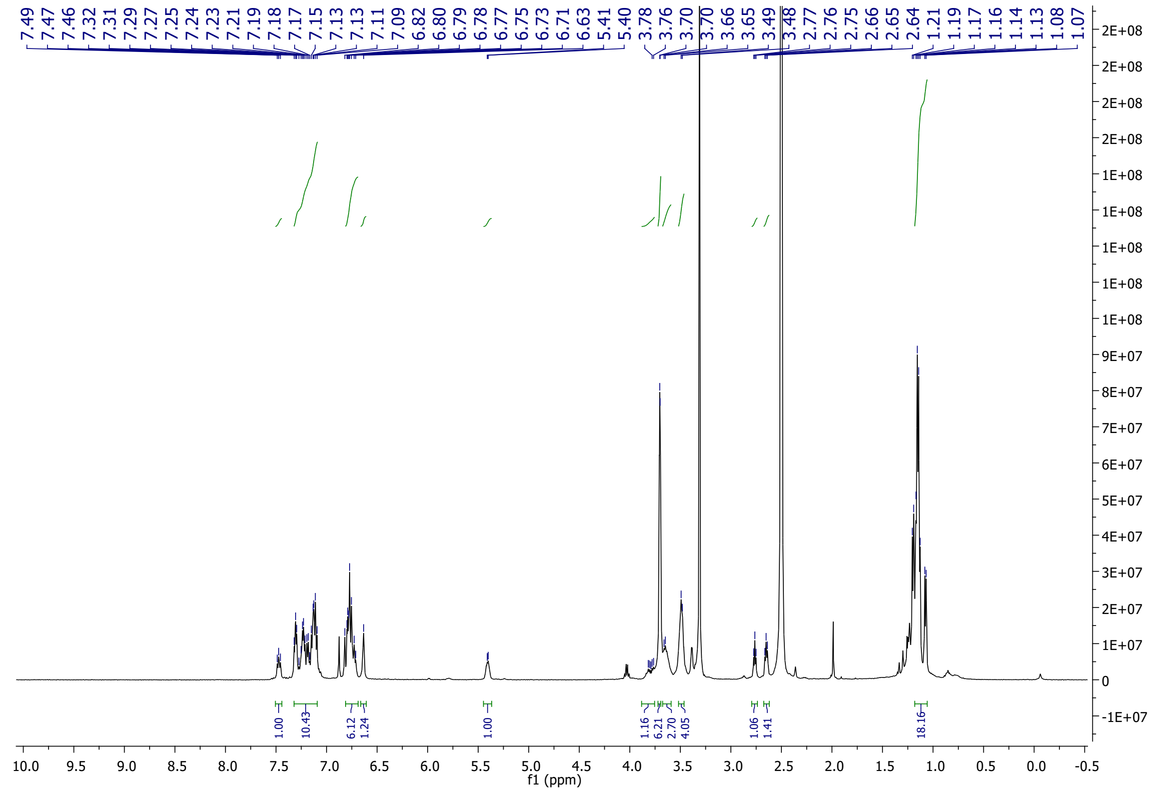

Figure S7: ^{1}H-NMR of 1-[2-(Dicyanomethylene)-7-(diethylamino)-2H-chromen-4-yl]-2-[(4-4'-dimethoxytrityl)oxy]ethyl(2-Cyanoethyl)diisopropylphosphoramidite in DMSO. The NMR shows residues of DCM and water.

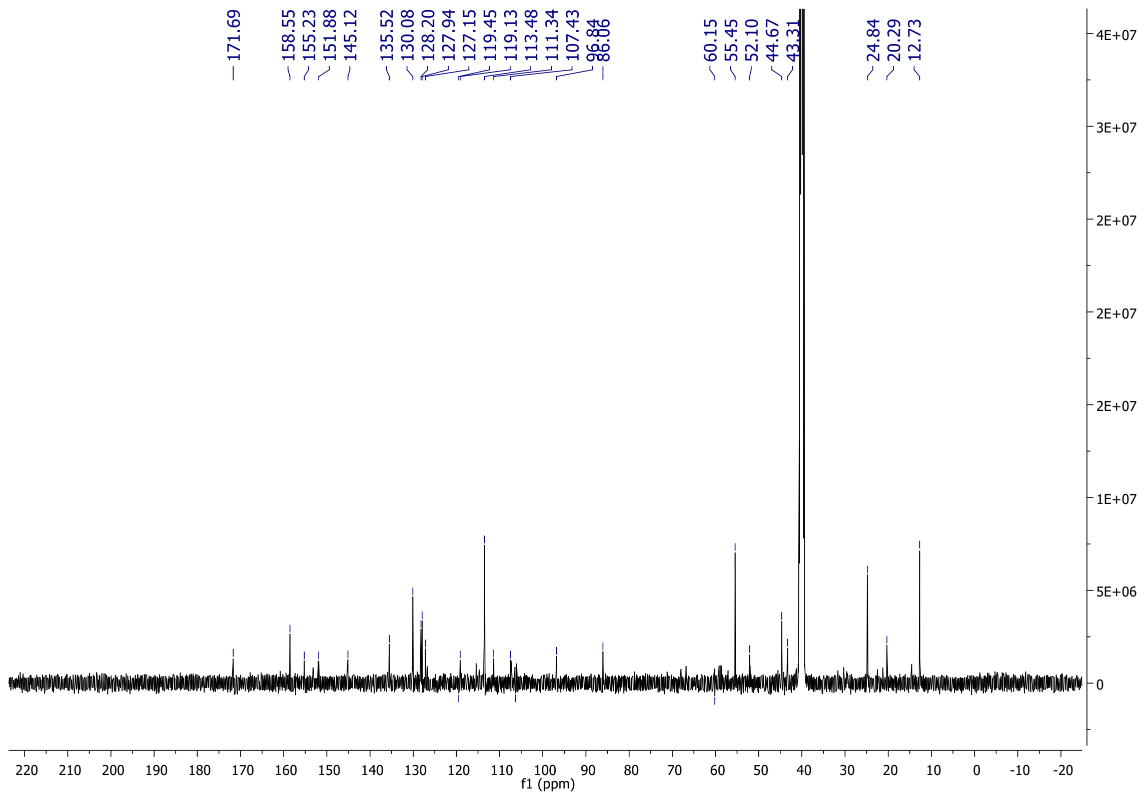

Figure S8: ^{13}C-NMR of 1-[2-(Dicyanomethylene)-7-(diethylamino)-2H-chromen-4-yl]-2-[(4-4'-dimethoxytrityl)oxy]ethyl(2-Cyanoethyl)diisopropylphosphoramidite in DMSO.

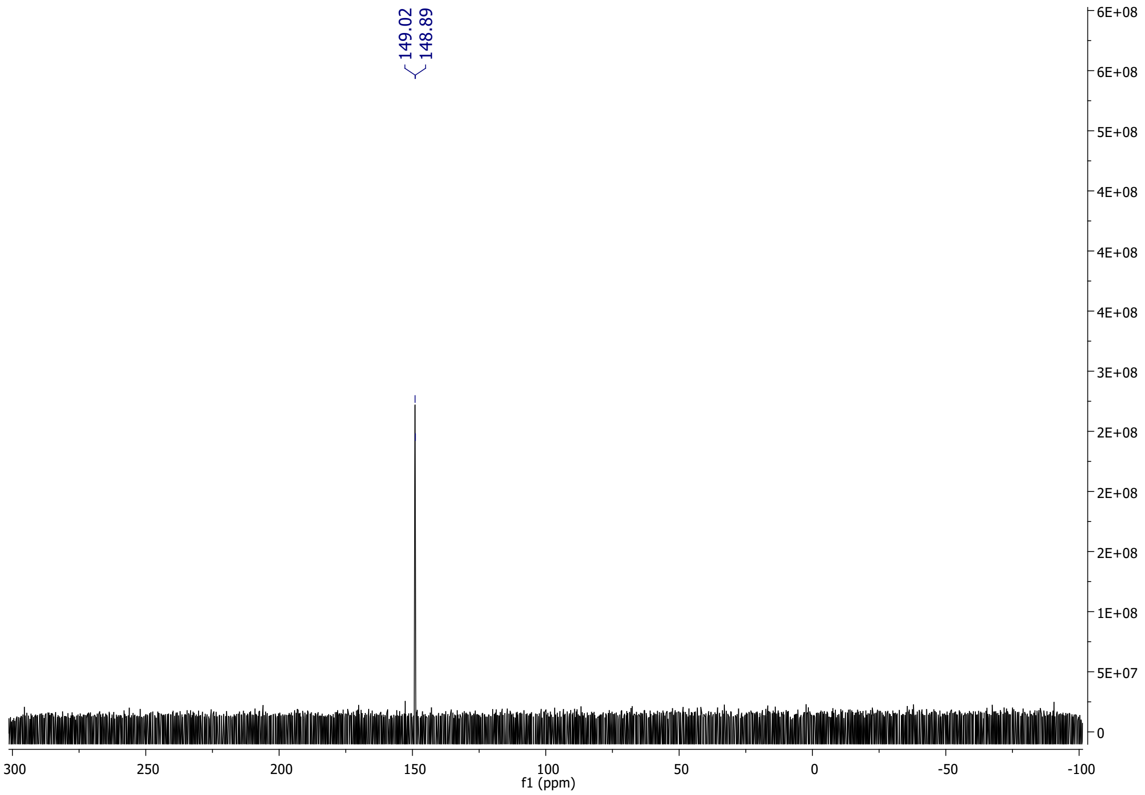

Figure S9 : ^{31}P-NMR of 1-[2-(Dicyanomethylene)-7-(diethylamino)-2H-chromen-4-yl]-2-[(4-4'-dimethoxytrityl)oxy]ethyl(2-Cyanoethyl)diisopropylphosphoramidite in DMSO.

6. Mass spectra of the oligonucleotides and photo products

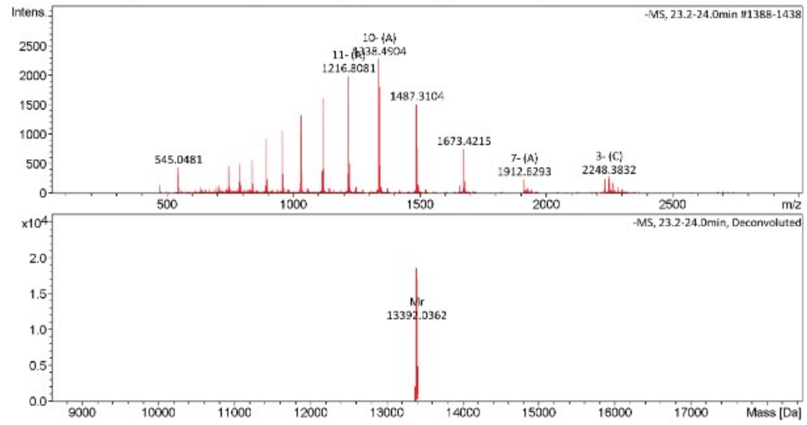

*Figure S10: Mass spectrum of **ON1**.*

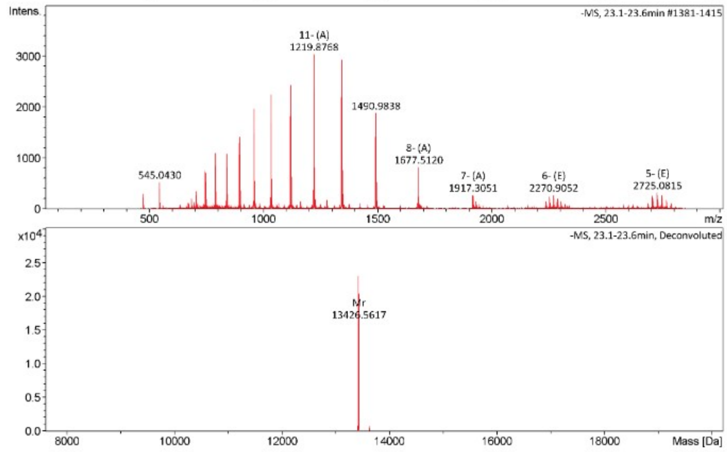

*Figure S11: Mass spectrum of **ON4**.*

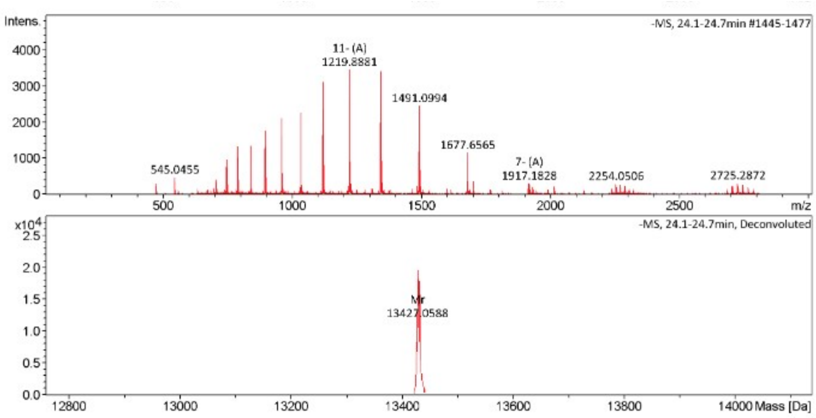

*Figure S12: Mass spectrum of **ON5**.*

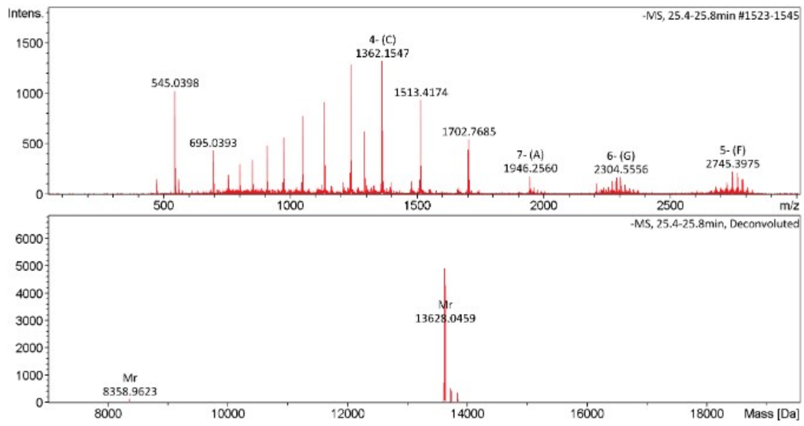

*Figure S13: Mass spectrum of **ON6**.*

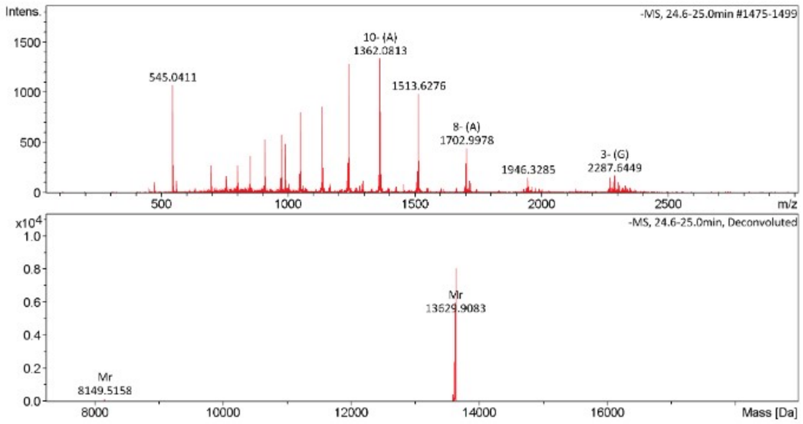

*Figure S14: Mass spectrum of **ON7**.*

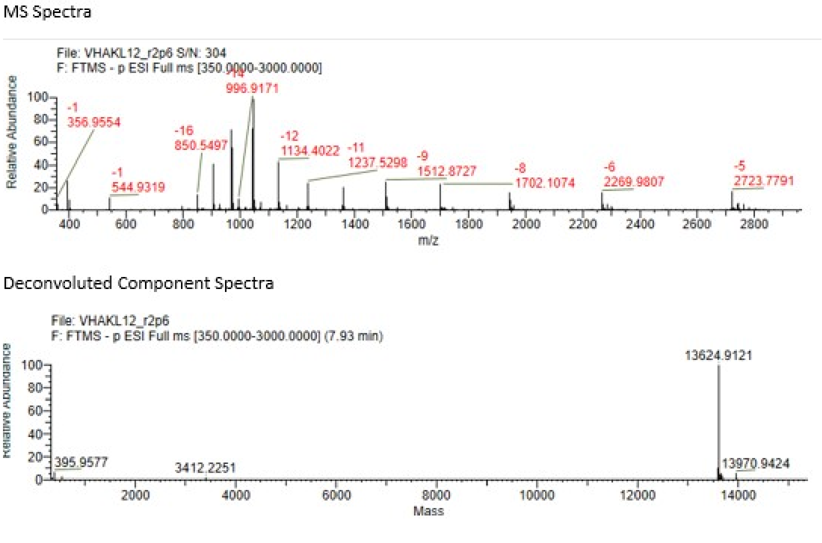

*Figure S15: Mass spectrum of **ON8**.*

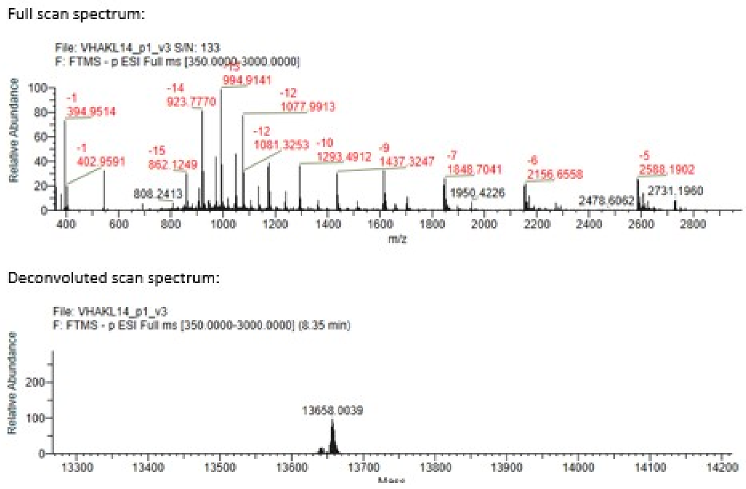

Figure S16: Mass spectrum of ***ON10****.*

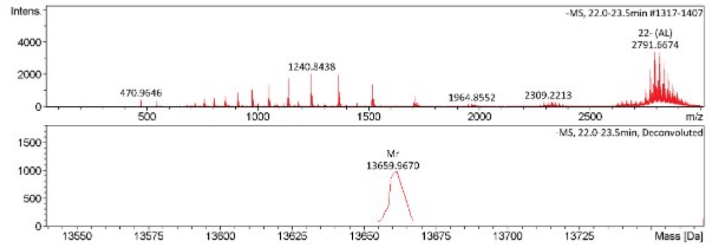

Figure S17: Mass spectrum of ***ON11****.*

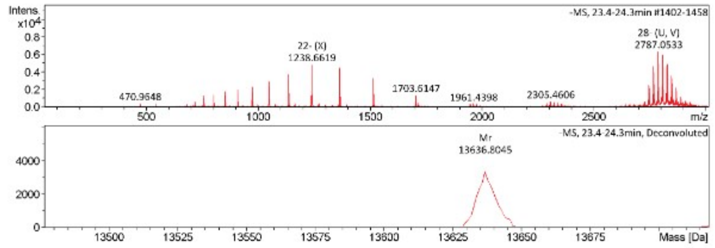

Figure S18: Mass spectrum of ***ON12****.*

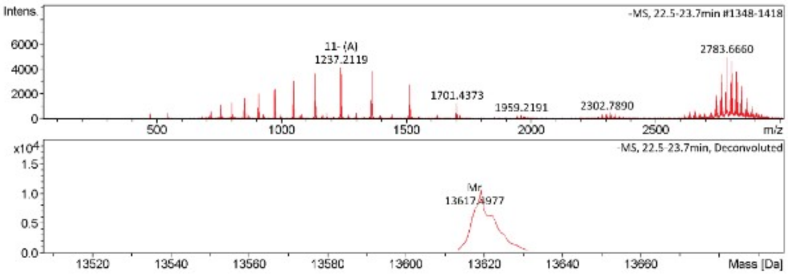

Figure S19: Mass spectrum of ***ON13****.*

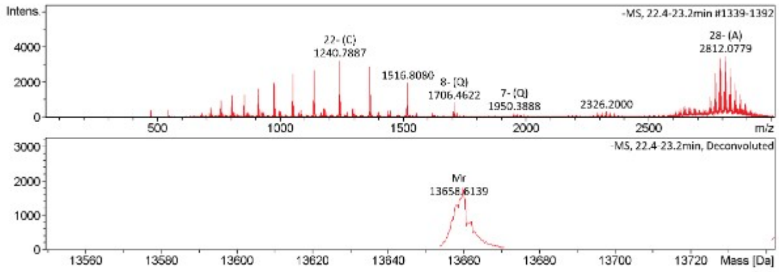

Figure S20: Mass spectrum of ***ON14****.*

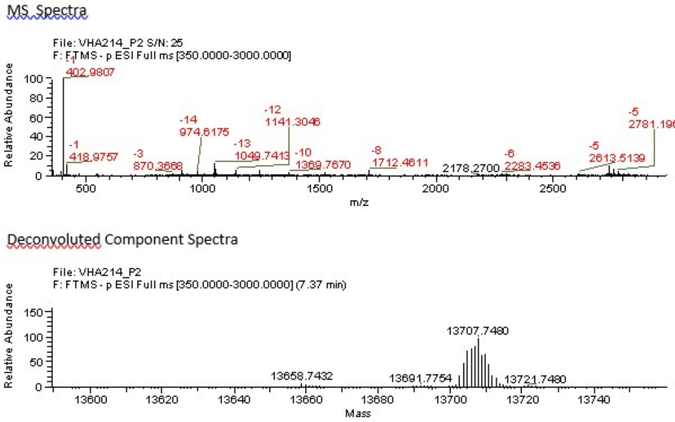

Figure S21: Mass spectrum of ***ON15****.*

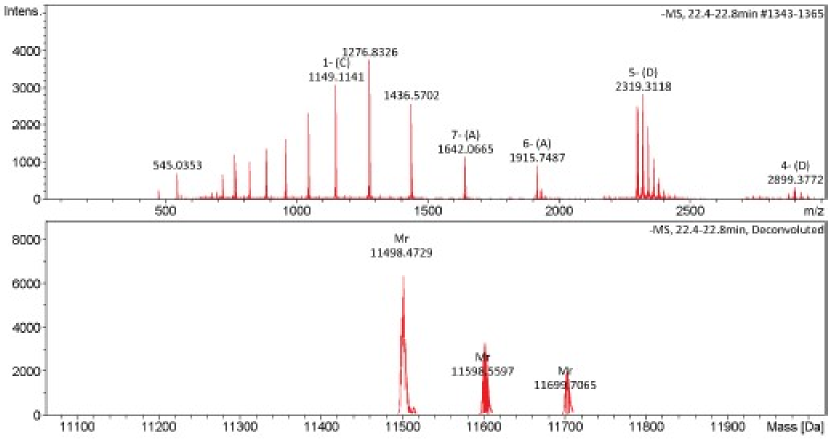

Figure S22: Mass spectrum of the according photo product of ***ON5****.*

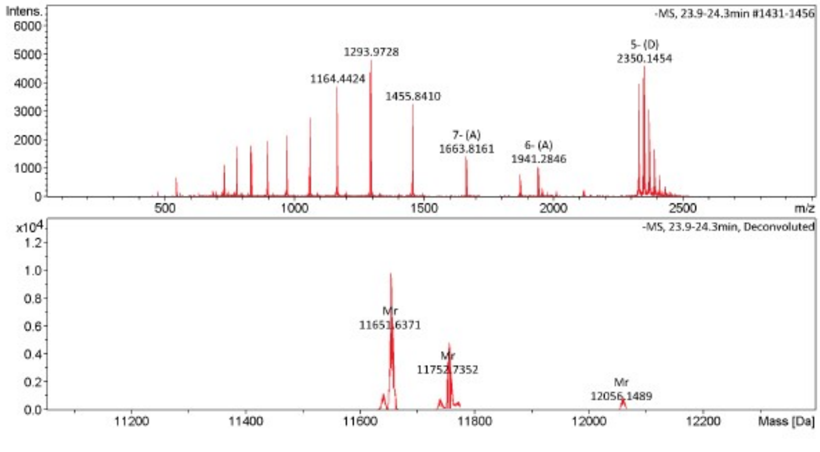

Figure S23: Mass spectrum of the according photo product of ***ON7****.*

7. Analytical RP-HPLC of oligonucleotides

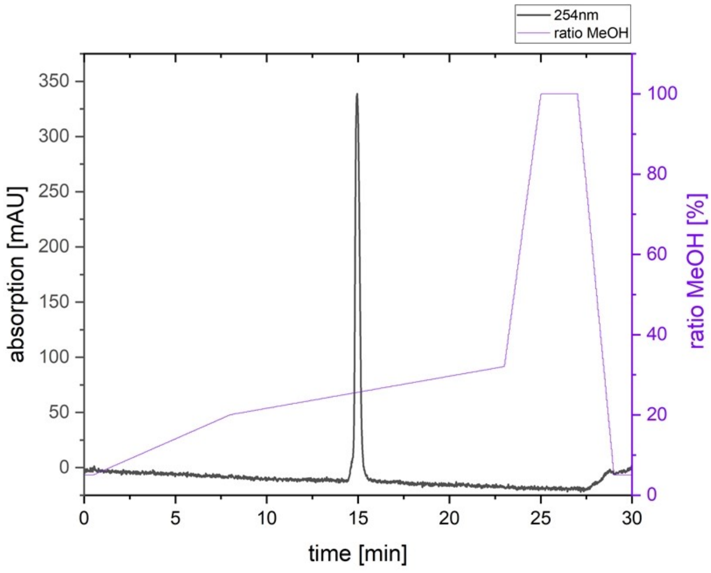

*Figure S24: Analytical RP-HPLC of 300 pmol **ON1**.*

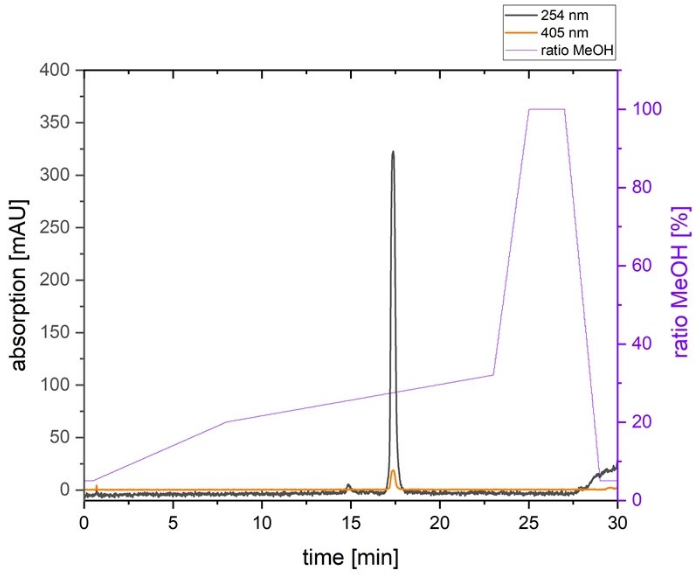

*Figure S25: Analytical RP-HPLC of 300 pmol **ON4**.*

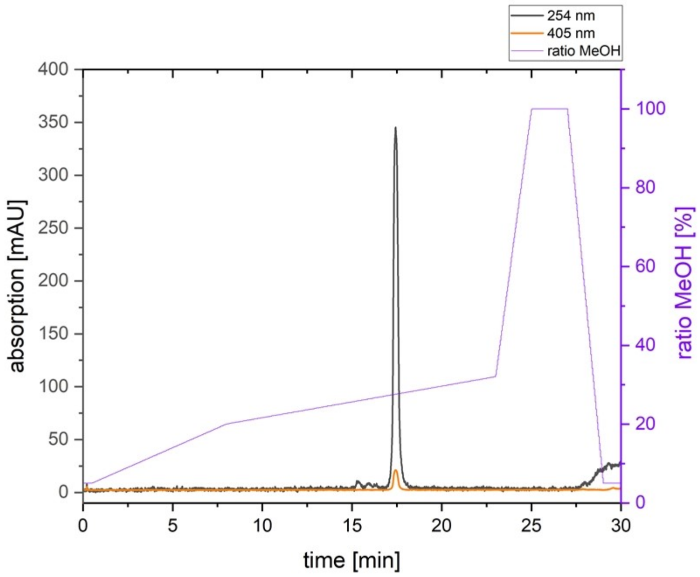

Figure S26: Analytical RP-HPLC of 300 pmol ***ON5***.

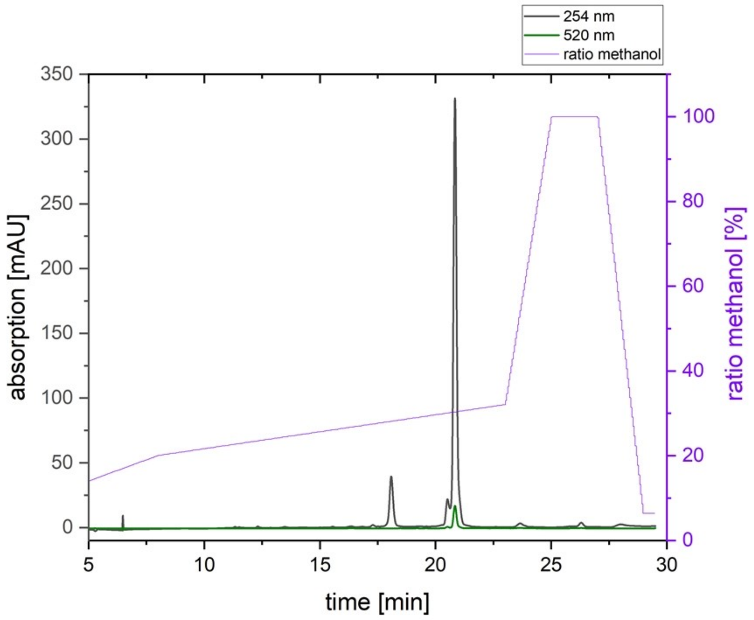

Figure S27: Analytical RP-HPLC of 300 pmol ***ON6***.

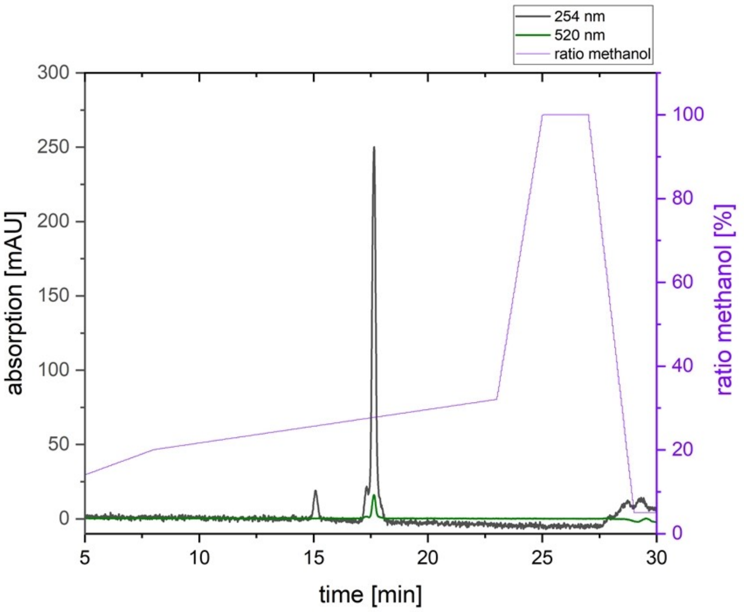

Figure S28: Analytical RP-HPLC of 300 pmol ***ON7****.*

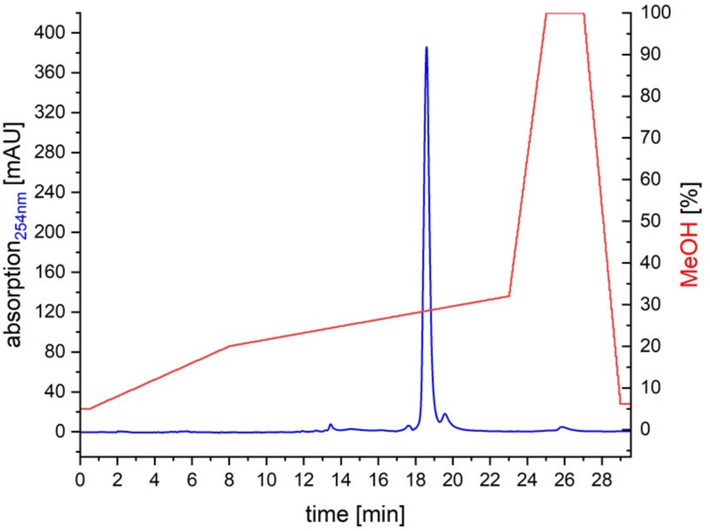

Figure S29: Analytical RP-HPLC of 300 pmol ***ON8****.*

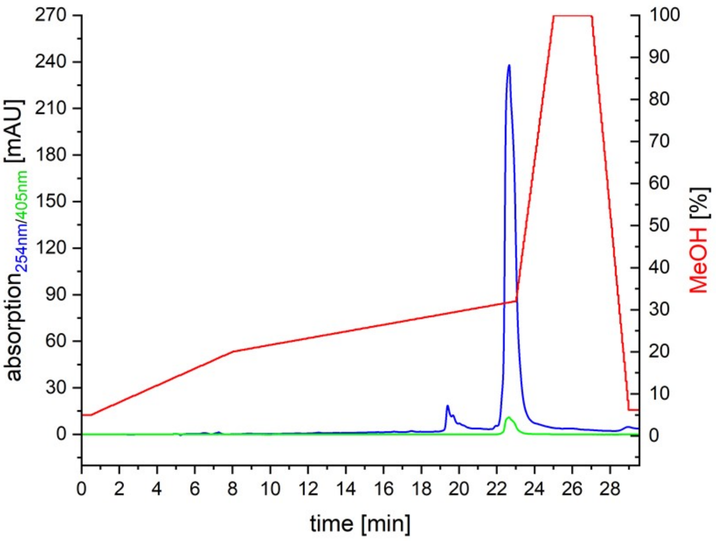

Figure S30: Analytical RP-HPLC of 300 pmol ***ON10****.*

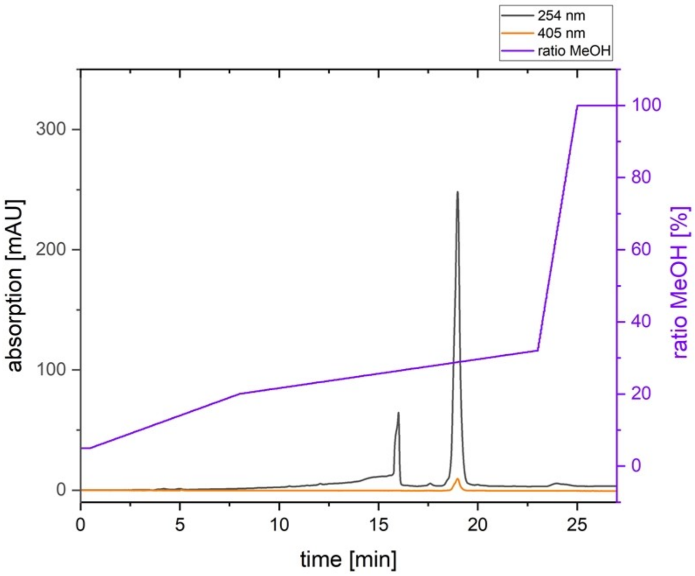

Figure S31: Analytical RP-HPLC of 300 pmol ***ON11****.*

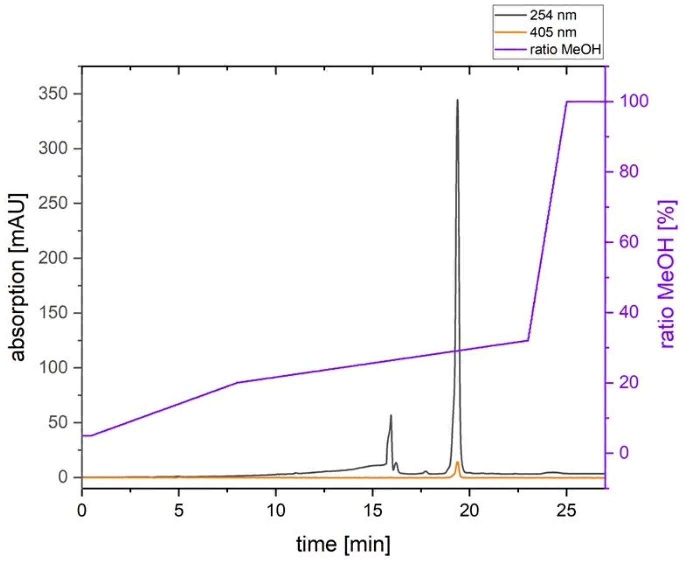

Figure S32: Analytical RP-HPLC of 300 pmol ***ON12****.*

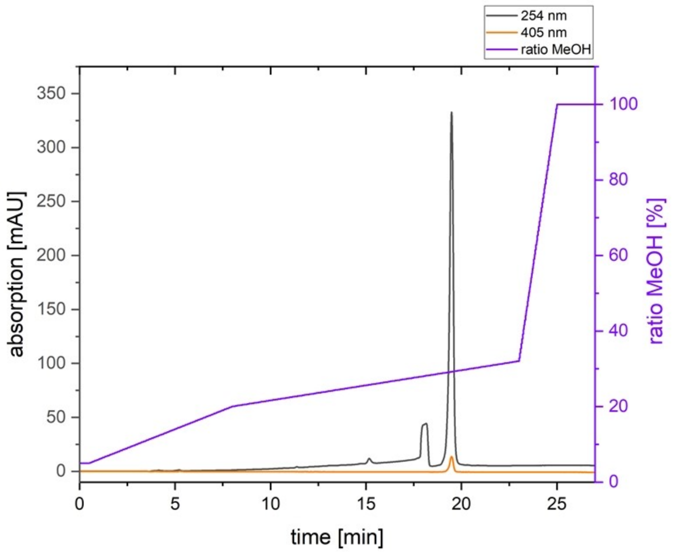

Figure S33: Analytical RP-HPLC of 300 pmol ***ON13****.*

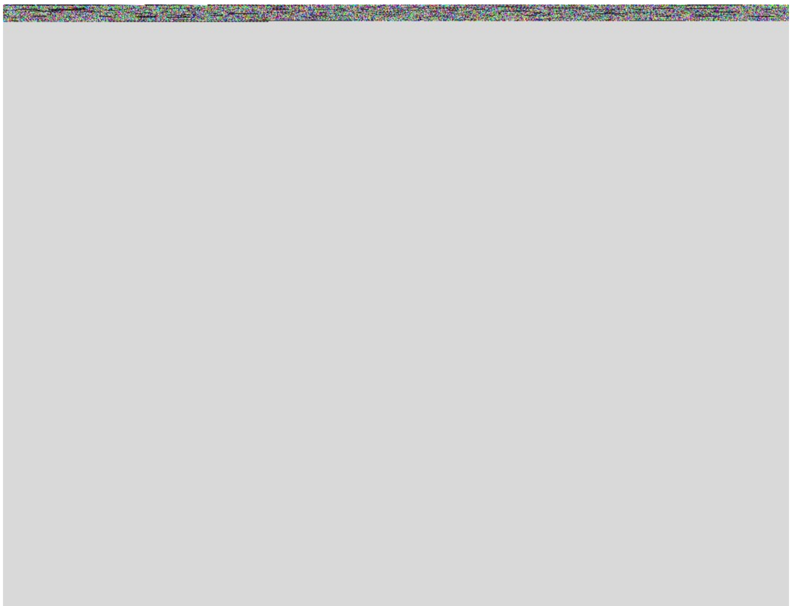

Figure S34: Analytical RP-HPLC of 300 pmol ***ON14****.*

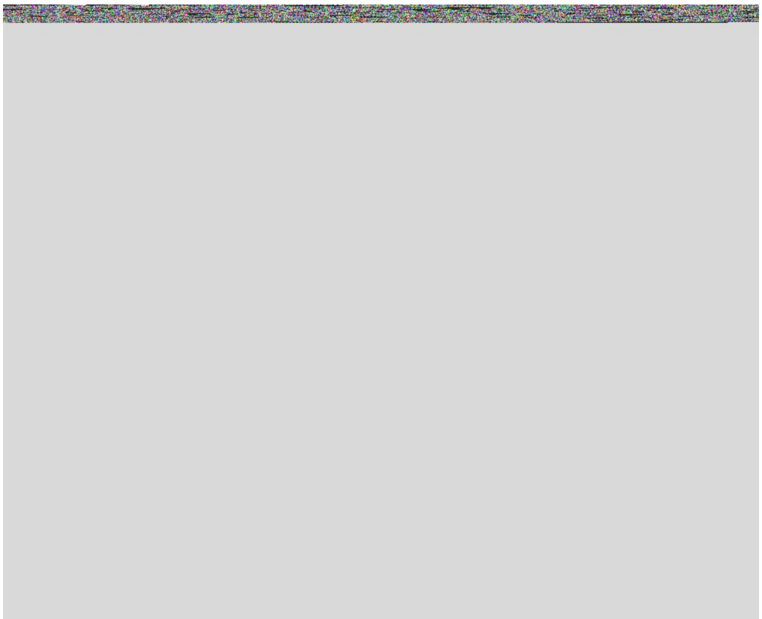

Figure S35: Analytical RP-HPLC of 300 pmol ***ON15****.*

8. References

[1] T. Weinrich, M. Gränz, C. Grünewald, T. F. Prisner, M. W. Göbel, *Eur. J. Org. Chem.* **2017**, *2017*, 491–496.

[2] X. M. M. Weyel, M. A. H. Fichte, A. Heckel, *ACS Chem. Biol.* **2017**, *12*, 2183–2190.

[3] J. Kaufmann, J. Wolf, A. Heckel, *Chem. - A Eur. J.* **2023**, *29*, e202300390.

[4] B. Schade, V. Hagen, R. Schmidt, R. Herbrich, E. Krause, T. Eckardt, J. Rgen Bendig, *J. Org. Chem.* **1999**, *64*, 9109–9117.

[5] R. W. J. Collin, K. W. Littink, B. J. Klevering, L. I. van den Born, R. K. Koenekoop, M. N. Zonneveld, E. A. W. Blokland, T. M. Strom, C. B. Hoyng, A. I. den Hollander, F. P. M. Cremers, *Am. J. Hum. Genet.* **2008**, *83*, 594–603.

[6] S. R. Hann, M. W. King, D. L. Bentley, C. W. Anderson, R. N. Eisenman, *Cell* **1988**, *52*, 185–195.

[7] F. Sinsel, M. Külp, M. A. Rieger, A. Heckel, *Chem. - A Eur. J.* **2025**, *31*, e202500347.
